# Genome-Wide Mapping Reveals an Extensive AtfA Regulatory Influence on Development, Metabolism, and Stress Preparedness in *Aspergillus nidulans*

**DOI:** 10.3390/cells14241965

**Published:** 2025-12-10

**Authors:** Márton Miskei, Sandugash Ibragimova, Beatrix Kocsis, Tibor Nagy, Hee-Soo Park, Tamás Emri, Jae-Hyuk Yu, Éva Leiter, István Pócsi

**Affiliations:** 1 HUN-REN-UD Fungal Stress Biology Research Group, University of Debrecen, 4032 Debrecen, Hungary; miskei.marton@science.unideb.hu (M.M.); sandugash.ibragimova@science.unideb.hu (S.I.); kocsis.beatrix@science.unideb.hu (B.K.); emri.tamas@science.unideb.hu (T.E.); 2 Department of Applied Chemistry, Institute of Chemistry, Faculty of Science and Technology, University of Debrecen, 4032 Debrecen, Hungary; nagy.tibor@science.unideb.hu; 3 School of Food Science and Biotechnology, Kyungpook National University, Daegu 41566, Republic of Korea; phsoo97@knu.ac.kr; 4 Department of Molecular Biotechnology and Microbiology, Institute of Biotechnology, Faculty of Science and Technology, University of Debrecen, 4032 Debrecen, Hungary; 5 Department of Bacteriology, University of Wisconsin-Madison, Madison, WI 53706, USA; jyu1@wisc.edu

**Keywords:** Aspergilli, asexual sporulation, primary metabolism, environmental stress response, bZIP-type transcription factor, oxidative stress, antioxidant defense, cell organelles, eisosome biogenesis, MAPK signaling cascade, light response, trehalose biosynthesis, polyol production, ChIP-seq, RNA-seq

## Abstract

**Highlights:**

**What are the main findings?**

- AtfA as a central transcription factor connects developmental and metabolic gene networks in *Aspergillus nidulans*; AtfA binding occurs constitutively, even under stress-free conditions, which is biologically meaningful and underexplored.
- AtfA is involved in the orchestration of MAPK signaling, eisosome assembly, redox homeostasis, expression of light-responsive proteins including transcription factors, and storage carbohydrate biosynthesis.

**What are the implications of the main findings?**

- The findings presented here advance our understanding of fungal biology across species, especially in how fungi preconfigure stress responses during conidiogenesis.
- Identifying the roles of AtfA adds multi-layered mechanistic depth to the field of study, and the new findings are highly relevant to the ecology, evolution, and pathogenesis of fungi.

**Abstract:**

Asexual sporulation (conidiogenesis) in filamentous fungi is a complex developmental process that requires precise coordination with primary metabolism and environmental stress responses. In the model fungus *Aspergillus nidulans*, we demonstrate that the bZIP-type transcription factor AtfA plays a central role in integrating conidiogenesis with the underlying metabolic and regulatory networks. Using combined ChIP-seq and RNA-seq analyses in wild-type, ∆*atfA*, and *atfA*-complemented strains under stress-free and oxidative stress (menadione) conditions, we identify a conserved AtfA binding motif and map its functional targets genome-wide. Our data reveal that AtfA binding to its target promoters is largely stress-independent, suggesting a preemptive regulatory mechanism in conidial development. AtfA directly binds to the promoters of genes involved in the MAPK signaling cascade, light-dependent sporulation, antioxidant defense, eisosome biogenesis, and the biosynthesis of trehalose and polyols—key metabolites supporting spore maturation and dormancy. Importantly, AtfA acts predominantly as a transcriptional activator, and its regulatory scope extends beyond stress adaptation to the orchestration of metabolic processes essential for spore integrity and germination. These findings position AtfA as a master integrator that synchronizes morphological development with metabolic preparedness during asexual reproduction in *A. nidulans*.

## 1. Introduction

Asexual sporulation (conidiogenesis in Ascomycete) is a fundamental developmental process in filamentous fungi, producing highly efficient, stress-tolerant propagules essential for survival and dissemination. Many conidia-forming fungi are of significant biomedical, agricultural, industrial, or environmental relevance, and accordingly, the molecular regulation of conidiogenesis has been extensively studied—particularly in the model organism *Aspergillus nidulans* [1,2]. This complex process involves the transition from vegetative hyphae to conidiophore formation, spore maturation, and, ultimately, dormancy and germination. Each step is tightly coordinated at the transcriptional level and supported by dynamic changes in cellular architecture and metabolism [3].

The generation of viable conidia requires extensive metabolic reprogramming to supply energy, reducing power, and biosynthetic precursors for the construction of cellular components, accumulation of storage molecules (e.g., trehalose, alditols), antioxidants, and protective pigments. These compounds not only ensure conidial longevity and stress resistance but also support rapid germination under favorable conditions [3,4,5]. Importantly, environmental stressors, particularly oxidative stress, further shape the molecular composition of conidia, thereby influencing their morphology, tolerance, and even virulence potential [6,7,8].

Among the stressors encountered by airborne conidia, molecular oxygen and its reactive derivatives (ROS) represent a persistent challenge. ROS are continuously generated by mitochondrial respiration and enzymatic activities, necessitating robust stress response systems. Conidiation, primary metabolism, and oxidative stress defense are therefore thought to be co-regulated, ensuring that conidia are developmentally mature and stress-protected by the time they are released [9,10].

The molecular regulation of conidiation has been most extensively characterized in *A. nidulans*, where genetic and transcriptomic studies have delineated both conserved and species-specific regulatory circuits [11,12,13,14]. This includes transcriptional cascades involving the central regulatory pathway (CRP), upstream developmental activators, and signaling networks responsive to environmental cues. Yet, how developmental processes like conidiogenesis are transcriptionally coordinated with primary metabolism and environmental stress responses remains largely unanswered.

One promising regulatory candidate is AtfA, a basic leucine zipper (bZIP)-type transcription factor orthologous to *Schizosaccharomyces pombe* Atf1. Members of this transcription factor family regulate diverse physiological processes, including development, stress responses, and secondary metabolism, across filamentous fungi [15]. In S. pombe, Atf1 plays a critical role in regulating oxidative and osmotic stress responses and also in coordinating sexual development [16,17,18]. Furthermore, Atf1 is essential for maintaining growth fitness under H_2_O_2_-induced oxidative stress, and Atf1 binding sites were constitutively present across the genome, independent of H_2_O_2_ treatment, suggesting a pre-established regulatory network [19]. Although most Atf1-bound genes were not transcriptionally induced by oxidative stress, they remained essential for stress resilience and cellular fitness [19]. Similarly to mammalian ATF/CREB transcription factors [20,21], fission yeast Atf1 recognizes a conserved palindromic cAMP response element (CRE) motif, 5′-TGACGTCA-3′, and this canonical motif is embedded within the broader Atf1-binding sequence 5′-ATGACGTCA-3′ [22].

In *A. nidulans*, AtfA has been shown to modulate oxidative and osmotic stress responses, with ∆*atfA* strains exhibiting hypersensitivity to oxidative agents and reduced viability of resting conidia [23,24,25]. Moreover, *atfA* deletion impairs cleistothecium formation and sterigmatocystin biosynthesis, suggesting broader regulatory roles [26]. In the related species *Aspergillus oryzae*, AtfA also governs conidial germination through the regulation of intracellular carbon and nitrogen stores, such as trehalose, alditols, and glutamate [27]. These findings collectively suggest that AtfA might act as a master transcription factor orchestrating development, metabolism, and stress adaptation.

Transcriptomic analyses have previously demonstrated that *A. nidulans* AtfA controls distinct gene sets depending on the developmental stage and stress conditions [4,5,26,28,29]. Interestingly, in conidia, AtfA appears to affect the expression of a largely invariant set of target genes regardless of oxidative stress exposure, suggesting that spores are pre-equipped for oxidative challenges and do not require further transcriptional adaptation post-development [5]. Among these target genes are key regulators of antioxidant defense, light responses, secondary metabolism, trehalose and glycogen metabolism, and conidial structure formation.

Among filamentous fungi, functional analysis of *S. pombe* Atf1 orthologs has been limited. To date, genome-wide binding analysis has only been reported for Aspergillus flavus AtfA. In this species, chromatin immunoprecipitation followed by sequencing (ChIP-seq) revealed that AtfA regulates genes involved in filamentous growth and responses to extracellular signals. However, the identified AtfA binding motif (5′-DRTGTTGCAA-3′ [30]) differs significantly from the canonical CRE sequence, suggesting divergence in DNA-binding specificity or regulatory context among Aspergilli.

In this study, we use ChIP-seq to identify functional AtfA binding sites in conidial genomes of *A. nidulans* harvested under both unstressed and menadione-induced oxidative stress conditions. By integrating these data with previous RNA-seq datasets, we comprehensively map the AtfA-dependent gene regulatory network during conidiogenesis. Our findings reveal that AtfA preemptively programs developing conidia with metabolic and structural resilience and position AtfA as a central transcription factor synchronizing asexual development with the primary metabolism and environmental defense systems that underpin fungal survival and dissemination.

**Key Advances of This Study**

1. First genome-wide ChIP-seq map of AtfA in any filamentous fungus.
2. Discovery that AtfA constitutively binds promoters under both stressed and unstressed conditions.
3. Identification of a conserved CRE-type motif identical to *S. pombe* Atf1, strengthening evolutionary links.
4. Integration of ChIP-seq, RNA-seq, metabolite profiling, and genetic phenotypes.
5. Direct transcriptional control of MAPK signaling, antioxidant defense, light-responsive factors, eisosome assembly, and carbohydrate storage metabolism.
6. Demonstration that AtfA pre-configures conidia for oxidative stress before environmental exposure.
7. Establishment of AtfA as a master integrator linking development, primary metabolism, and stress resilience.

## 2. Materials and Methods

### 2.1. ChIP-Seq Analysis

*atfA*::3XFLAG (*pyrG89*; *pyroA4*; Δ*atfA*::*AfupyrG^+^*; *atfA*-3XFLAG::*pyroA^+^*; *veA^+^*) strain for ChIP analysis was constructed with the complementation of Δ*atfA* (*pyrG89*; *pyroA4*; Δ*atfA*::*AfupyrG^+^*; *veA^+^*) with pHS13 vector carrying *atfA*::3XFLAG sequence with *atfA*’s native promoter as well as *trpC* terminator of *A. nidulans*. The *atfA* deletion mutant was constructed by double-joint PCR (DJ-PCR) [4]. *atfA*-3XFLAG complemented mutants were checked genotypically by confirming the integration of the promoter-*atfA*-3XFLAG into Δ*atfA* genome with PCR and phenotypically by verifying the stress sensitivity of the mutants with comparable phenotypes as that of the wild-type strain (Figure S3). For sample preparation, 2 d- and 3 d old untreated and 3 d old 0.04 mM MSB (menadione sodium bisulfite; increasing intracellular superoxide level)-treated conidia [5] (~2 × 10^9^ conidia) from surface cultures grown on NMM agar plate [4] from the strain *atfA*::3XFLAG were cross-linked with freshly prepared 1% formaldehyde at room temperature for 30 min. Following that, 1/20 volume of 2.5 M glycine solution was added to stop the cross-linking reaction. Conidia were then collected by centrifugation (5000× *g*, 5 min, 4 °C) and washed with ice-cold PBS three times. About 1.5 mL FA lysis buffer {50 mM HEPES-KOH [pH 7.5], 150 mM NaCl, 1 mM EDTA, 1% Triton X-100, 0.1% Na deoxycholate, 0.1% SDS, and 1 protease inhibitor cocktail tablet (Roche) per 50 mL} was added before use. Glass beads were added into cross-linked cell lysates, which were broken by a mini-bead beater (MagNa Lyser instrument, Roche, Basel, Switzerland) for three cycles (1 min homogenization followed by 1.5 min resting on ice). Subsequently, the samples were sonicated for eight cycles (60 s on, 60 s off) with a sonifier level 5 of output control in a Diagenode Bioruptor (Diagenode, Denvill, NJ, USA). All steps were carried out at 4 °C. The sonicated cell lysates were cleared of cellular debris by centrifugation at 13,000 rpm for 10 min at 4 °C. The supernatant was collected and subjected to chromatin IP with mouse monoclonal Anti-Flag M2 antibody (Merck, Rahway, NJ, USA) using MAGnify™ Chromatin Immunoprecipitation System (Invitrogen, Carlsbad, CA, USA) following the manufacturer’s protocol.

ChIP-Seq libraries were prepared with Ovation Ultralow Library Systems V2 (Tecan, Mannedorf, Switzerland) according to the manufacturer’s instructions. Briefly, 10 ng of immunoprecipitated DNA was used for library generation. Repairing end step was followed by indexed adaptor ligation and enrichment of adaptor ligated fragments by 10 PCR cycles. Fragment size distribution of the libraries was checked on BioAnalyzer 2100 (Agilent Technologies, Santa Clara, CA, USA) using DNA High Sensitivity chip. Single-read 75 bp sequencing reads were generated by NextSeq 500 sequencer (Illumina, San Diego, CA, USA). Library preparations and sequencing were performed by the Genomic Medicine and Bioinformatics Core Facility of the University of Debrecen.

### 2.2. AtfA Binding Motif Analysis and Comparison of ChIPseq and RNAseq Data

ChIP-seq experiments were performed in two replicates per culture condition. After quality control of the sequenced data, reads were aligned to the appropriate *Aspergillus nidulans* reference genome [31,32] (https://mycocosm.jgi.doe.gov/Aspnid1/Aspnid1.home.html, accessed on 24 September 2025) with Bowtie2 (version 2.2.4) [33] (ChIP-seq) softwares. The Picard toolkit (Broad Institute, GitHub repository, https://broadinstitute.github.io/picard/; version 3.4.0, accessed on 13 April 2025) removes PCR duplicates from BAM files created by the SAMtools [34] package. RPKM normalization is performed by deeptools [35]. In ChIP-seq analysis, MACS2 [36] determines peaks in bedGraph files with the default parameters. The obtained binding sites (BED files) were used for further analyses. Using bedtools [37], the corresponding sequences were extracted from the *A. nidulans* reference genome. These sequences were then analyzed with MEME Version 5.5.8 (“Multiple Expectation Maximization for Motif Elicitation”) [38] to identify overrepresented sequence motifs. The occurrence of the identified motifs across the entire genome was assessed using the FIMO algorithm [39]. ChIP-seq data were deposited in the National Center for Biotechnology Information (NCBI) databases like GEO (http://www.ncbi.nlm.nih.gov/geo/, accessed on 29 August 2025), TSA (https://www.ncbi.nlm.nih.gov/genbank/tsa/, accessed on 29 August 2025).

To evaluate the overlap between AtfA binding sites identified under different experimental conditions, we used the R programming environment. Sets of ChIP-seq peaks from untreated and MSB-treated samples were compared.

The genomic localization and enrichment of the ChIP-seq peaks were also assessed using bedtools [37] and R. For each condition, we generated a set of random binding sites matching the number of observed peaks. Then, the frequency of observed vs. random peaks was compared in various genomic regions, including 3′UTRs, 5′UTRs, CDSs, and regions around transcription start sites (TSS-500, TSS-1500, and TSS+50-1000). Our preliminary analyses already revealed the approximate distribution range of the reads and their typical localization relative to the TSS, where we used computeMatrix commands of the deepTools package [35]. Enrichment or depletion in these regions was tested using a Z-test (R packages; version 4.5.1).

Genes with AtfA binding sites in their putative promoter regions were identified under all experimental conditions tested in this study (in both unstressed and MSB-exposed cultures). Changes in expressions of AtfA-regulated genes recorded previously in RNA-seq-based transcriptomics studies by Kocsis et al. (2023) [5] (GEO accession number: GSE220052) were compared to those of randomly selected gene sets of equal size from the *A. nidulans* proteome using a Wilcoxon test in R.

To gather further information about their physiological functions related to environmental stress response, we also searched for the *Aspergillus nidulans* Stress Protein Database updated version 2024 for *A. nidulans* genes that have at least one active AtfA binding site in their promoters. (Table S1). A manual literature search was performed as before [40,41], and this database now incorporates 342 functionally characterized stress proteins with their annotation (Table S1).

AtfA ChIP-seq results associated with gene expression changes in *A. nidulans* Δ*atfA* and wild-type strains [5] (Table S2), description of *A. nidulans* genes possessing *AtfA*-bound promoters showing altered expression in Δ*atfA* compared with wild-type (Table S3), and annotation of genes {FungiDB database (FungiDB-68_AnidulansFGSCA4.gff, https://fungidb.org/fungidb/app/downloads, accessed on 27 May 2024, release 68), *Aspergillus nidulans* Stress Protein Database (Table S1)} are available in the literature, databases, and Supplementary Tables Files as indicated in parentheses. Gene lists available in Supplementary Tables Files were manually curated and were simplified as required and as indicated in Supplementary Tables File Sheet descriptions.

AN identifiers of *A. nidulans* genes with AtfA binding sites in their promoters were retrieved from the second worksheet of Table S3 (Sheet 2, containing a total of 502 genes, is a manually curated version of the dataset presented on Sheet 1). GO enrichment analysis was then performed using the GO Enrichment tool available on FungiDB (https://fungidb.org/fungidb/app, accessed on 27 May 2024, release 68). Categories with significant enrichment (FDR < 0.05) were summarized in Table S4. A selected group of GO terms related to the study were also displayed in a bubble chart in Figure S4.

### 2.3. Conidia Yields and Analytical Data

The conidiospore-forming capabilities of untreated and MSB-treated cultures of the *atfA*::3XFLAG strain were determined as published by Kocsis et al. (2022) [26]. Briefly, conidia from 2- and 3-day-old cultures were washed, counted in a Bürker chamber, and spore numbers were expressed as number/plate. Glucose concentrations in the 2 d and 3 d untreated and 0.04 mM MSB-treated surface cultures from the *atfA*::3XFLAG strain were determined using a modified colorimetric enzymatic method based on glucose oxidase and peroxidase activity, as described elsewhere [42,43]. Briefly, a 10 mm × 10 mm piece of agar with fungal cultures was cut using a sterile scalpel and divided into two equal parts. Fungal cultures were kept on agar pieces. Then, these two agar pieces were placed (separately) into Eppendorf tubes with 0.5 mL of phosphate buffer (13.5 g Na_2_HPO_4_ 2H_2_O/L, pH 6.6) and incubated at 4 °C for 30 min, and the glucose concentration of the liquid phase was determined according to Leary et al. (1992) [43]. Trehalose, erythritol, glycerol, and mannitol content of conidiospores were determined according to Suzuki et al. (2013) [44]. Briefly, 2 × 10^8^ conidia were dissolved in 300 μL distilled water and heat treated at 98 °C for 3 h. Supernatant was collected by centrifugation at RT, at 16,000× g for 20 min, and subjected to HPLC analysis. The HPLC-MS measurements were performed by a Waters 2695 Separation Module with a thermostable autosampler (5 °C) and a column module (35 °C) (each from Waters, Milford, MA, USA). An Aminex HPX-87C column (7.8 × 300 mm) was used with isocratic elution. The mobile phase was water (Direct-Q water system (Millipore, Molsheim, France) with a flow rate of 0.3 mL/min. An amount of 50 µL was injected from each sample.

For the mass spectrometric detection, a MicroTOF-Q type Qq-TOF MS instrument (Bruker Daltoniks, Bremen, Germany) equipped with an ESI ion source was used in positive ion mode. The spray voltage was 4 kV, while nitrogen was used as drying (200 °C) and nebulizing gas (1.6 bar). All measurements were recorded using a digitizer at a sampling rate of 2 GHz. The mass spectra were calibrated externally using the ESI-tune mix from Bruker.

The glycerol, erythritol, and mannitol were detected mainly in the form of sodiated adduct ions [M+Na]^+^, while the trehalose was detected in different forms. Due to the presence of Ca^2+^ ions in the column, Ca^2+^ ion adducts and clusters were detected: [M+Na]^+^, [2M+Ca]^2+^, [3M+Ca]^2+^, [4M+Ca]^2+^, [5M+Ca]^2+^, and [6M+Ca]^2+^. All the adducts of the trehalose were used. For quantification, extracted ion chromatograms were applied.

Conidiospore yields, trehalose, erythritol, glycerol, and mannitol content of conidiospores, as well as remaining glucose concentrations, were determined in three independent experiments and are presented as mean ± SD values. Statistical significances were calculated using Student’s *t*-test, and *p*-values less than 5% were considered as statistically significant.

## 3. Results and Discussion

### 3.1. Sampling and Physiological Characterization of Conidia

As presented in Table 1, conidia yields were the same in 3 d unstressed and stress-exposed cultures, and the starting glucose had already been completely used up. Because the same conidia yields with no remaining glucose in the agar medium had also been reached at 2 d incubation time in unstressed cultures, conidia from all these cultures were included in ChIP-seq analysis {Samples Untr. (2 d), Untr. (3 d), MSB Tr. (3 d)}. Considering intracellular reserves, trehalose and mannitol abundances were recorded in Untr. (3 d) and MSB Tr. (3 d) spores, which is in line with previous observations [44,45] (Table 1). It is worth noting that MSB Tr. (3 d) spores accumulated more mannitol; meanwhile, Untr. (3 d) conidia contained more trehalose, and 3 d conidia contained significantly more trehalose and mannitol than 2 d spores, except for the mannitol content of MSB Tr. (2 d) and (3 d) conidia, which were equal (Table 1). Erythritol and glycerol were produced typically at low concentrations and were more detectable in conidia harvested from 2 d cultures (Table 1).

### 3.2. ChIP-Seq Data Generation and Processing

#### 3.2.1. Motif and Localization of *A. nidulans* AtfA Binding Sites

ChIP-seq peaks {Untr. (2 d): rep1: 473, rep2: 434, Untr. (3 d): rep1: 473; rep2: 451, MSB Tr. (3 d): rep1: 453; rep2: 439; NCBI GEO identifier: GSE306765} were mapped to the *A. nidulans* reference genome to assess their overlaps, which were as high as those observed between biological replicates under the same experimental conditions (mean overlap was ≈77%; Table 2). AtfA bound to the great majority of the identified binding sites independently of MSB treatments, greatly supporting the idea that *A. nidulans* conidia did not have to adapt to MSB-elicited oxidative stress under these experimental conditions [5]. This observation is in good accordance with previous ChIP-chip data gained in H_2_O_2_-exposed *S. pombe* cells, where Atf1 binding sites were constitutively present even before H_2_O_2_ stress [19].

**Figure 1:**
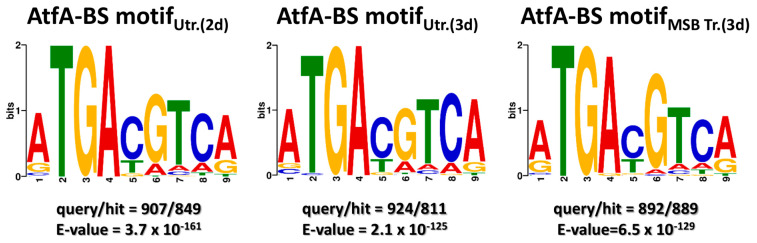
**Consensus binding motifs of AtfA.** Consensus motif sequences were identified by MEME motif discovery based on ChIP-seq peak sequences. ChIP-seq was performed on untreated 2-day-old and 3-day-old spores, as well as on 3-day-old spores treated with MSB. Query and hit numbers represent the total number of peaks and the number of peaks containing the motif sequence, respectively. E-value represents the expected number of motifs of equal or greater statistical significance that could be found in random sequences of the same size.

Unlike in the case of *A. flavus* AtfA [30], the consensus *A. nidulans* AtfA binding motif (5′-ATGACGTCA-3′, independently of the culture conditions; Figure 1) was identical with the Atf1 binding motif in fission yeast (5′-ATGACGTCA-3′, containing the palindromic canonical CRE sequence 5′-TGACGTCA-3′ [22]), which is consistent with our previous finding that *A. nidulans atfA* is a true functional ortholog of *S. pombe atf1* [24].

**Figure 2:**
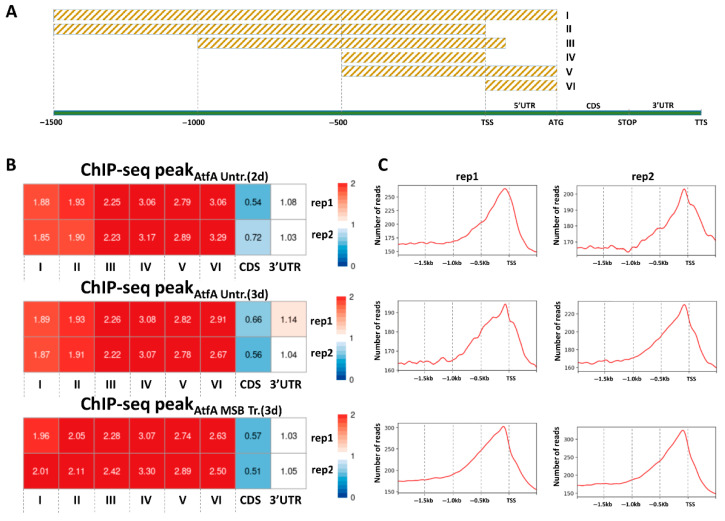
**Localization of AtfA ChIP-seq peaks under different conditions, by replicate.** (**A**) Roman numerals indicate upstream regions in which we compared the occurrence of observed peaks and random peaks. (**B**) Enrichment/depletion of peaks in these regions (red indicates significant enrichment by proportion test, *p* < 0.001; blue indicates significant depletion). (**C**) Distribution of ChIP-seq reads across the upstream regions of all protein-coding genes.

Considering the distribution of AtfA ChIP-seq peaks across promoters and coding-region neighborhoods (Figure 2), 86.7% of the detected peaks on average fell within these regions. ChIP-seq peaks were significantly (*p* < 0.001) enriched in the 5′ UTR (5′ untranslated region) and upstream of the transcription start site (TSS) compared to randomly generated peaks (Figure 2B). In contrast, peaks were significantly depleted in coding sequences (CDS), while no difference was observed in the 3′ UTR (3′ untranslated region) relative to random peaks (Figure 2B). When the localization of ChIP-seq reads was visualized within the examined regions, the peak summit was positioned upstream of the TSS, with the majority of reads located within the −500 bp–TSS region. The wider −1000 to +50 bp from the TSS interval encompassed the entire peak marked by ChIP-seq reads in the promoters of protein-encoding genes (Figure 2C and Figure S1, Table S2), supporting the view that AtfA is a true transcription factor similar to *S. pombe* Atf1 [22]. For the subsequent analyses, we used both the narrower TSS–500 region and the broader −1000 to +50 bp window around the TSS [46]. The TSS–500 interval provided the strongest enrichment of AtfA binding sites compared to random controls, whereas the extended −1000/+50 bp region allowed the detection of additional, more distal binding sites on both sides of the TSS [46].

MEME-defined AtfA binding motifs were mapped across the entire genome using the FIMO algorithm, yielding a total of 7127 motif instances combined from the data coming from the three experimental arrangements tested, and 72.1% of genes with a ChIP-seq peak in the region spanning the −1000 to +50 bp from TSS interval also contained at least one AtfA binding motif within the same region. In many cases, paired binding motifs were observed reflecting the potential of AtfA to regulate target genes as a homodimer or a heterodimer formed with another bZIP-type transcription factor, which also recognizes the canonical CRE sequence like Pcr1 in *S. pombe* [19,22].

The consensus binding motif of AtfA (5′-ATGACGTCA-3′) contains the palindromic canonical CRE sequence 5′-TGACGTCA-3′ [20,21,22]; therefore, we assumed that AtfA could bind both DNA strands with comparable affinity. Hence, the orientation of the binding motif was disregarded by us in the analysis of upstream regions (upstream of TSS, 5′ UTR). This approach has been further justified by recent studies systematically investigating motif orientations and reaching the conclusion that orientation determines, only in relatively few cases, whether or not a binding site is functional, particularly in the case of palindromic or near-palindromic sequences [47,48,49].

#### 3.2.2. Expression Changes in Genes with AtfA Binding Sites

**Figure 3:**
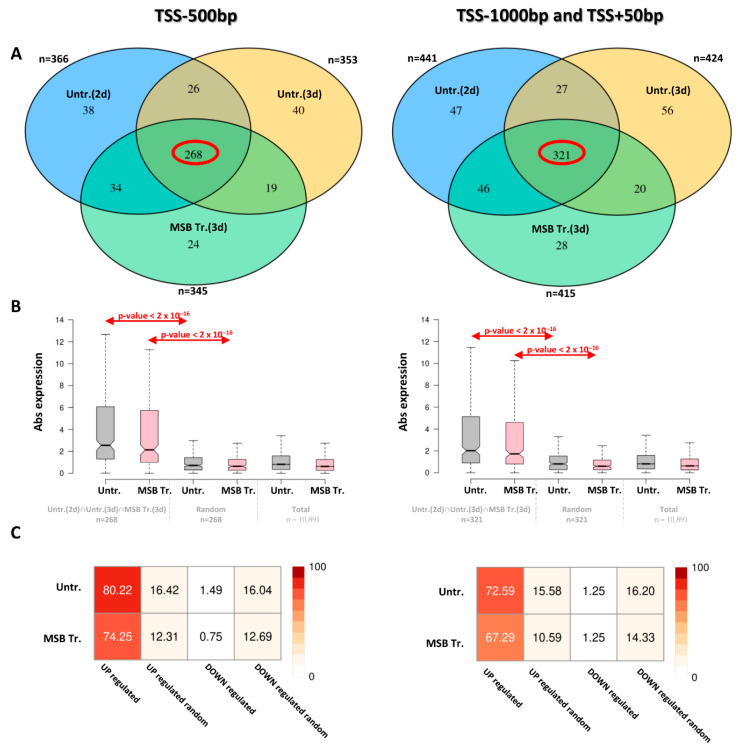
**Expression changes between Δ*atfA* vs. wild-type strains for genes harboring AtfA ChIP-seq peaks.** (**A**) Protein-encoding genes with AtfA peaks identified in all three samples. The red ellipses indicate the number of protein-coding genes present in the intersection shared by all three analyses. (**B**) Absolute expression changes in genes with AtfA ChIP-seq peaks identified under all three conditions [Untr. (2 d), Untr. (3 d), and MSB Tr. (3 d)]. RNA-seq was performed on 3-day-old untreated and menadione-treated spores from both wild-type and Δ*atfA* strains. We also examined absolute expression changes in a random set of genes and across the entire transcriptome. (**C**) Percentage of up- and downregulated genes with AtfA peaks in 3-day untreated and menadione-treated samples, compared with randomly selected gene sets.

All protein-encoding genes with an AtfA ChIP-seq peak in their upstream regions (upstream of TSS, 5′ UTR) were compiled independently of culture conditions, and the total numbers of loci with an AtfA ChIP-seq peak in their −500 bp–TSS and −1000 to +50 bp from TSS regions were 449 and 545, respectively (Table S2). Importantly, as many as 268 and 321 loci (Table S2) were detected in the −500 bp–TSS and −1000 to +50 bp from TSS regions, respectively, which were recognized by AtfA in at least one replicate under each of the three culture conditions (2-day unstressed, 3-day unstressed, and MSB-treated) (Figure 3A). In both sets of these overlapping loci, the occurrences of significant gene expression changes (upregulated and downregulated combined, 3 d conidiospores [5]) were significantly higher than in either randomly selected gene sets or in the full genomic gene set (Wilcoxon test, *p* < 0.001; Figure 3B,C). Moreover, genes associated with an AtfA ChIP-seq peak were predominantly upregulated in the presence of functional AtfA (Figure 3C); this effect was not observed in size-matched random gene sets. This observation clearly indicates that AtfA is a positive-acting transcriptional regulator with a few exceptions, which is in line with the data gained previously with *S. pombe* Atf1 [19].

### 3.3. AtfA Is Involved in the Regulation of Conidiogenesis

Unless otherwise indicated, all data discussed in this section are summarized in Table S3, Sheet 6 (Title: “*A*. *nidulans* genes with AtfA-bound promoter”, Subtitle: “*A. nidulans* genes possessing AtfA-bound promoters showing altered expression in Δ*atfA* compared with wild-type”). Note that this dataset was manually curated, and any genes whose expression was not affected by *atfA* deletion under either stressed or non-stressed culture conditions (NA/NA genes) were excluded from further analysis. Genes of unknown function (FUN), which refer only to FUN genes found in other *Aspergillus* species, were also ignored, and gene functions were updated with the latest literature data (Table S3, Sheets 1–6). This study focused primarily on mapping genes positively regulated by AtfA in either unstressed or MSB-exposed cultures or both; therefore, mechanisms that eliminate the effects of *atfA* gene deletion on some AtfA-target genes with the AtfA-bound promoter (Table S3, Sheets 1 and 2) are not analyzed in this article, but, in some cases, likely regulatory pathways that counteract the positive effects of AtfA on gene expression are discussed.

**Figure 4:**
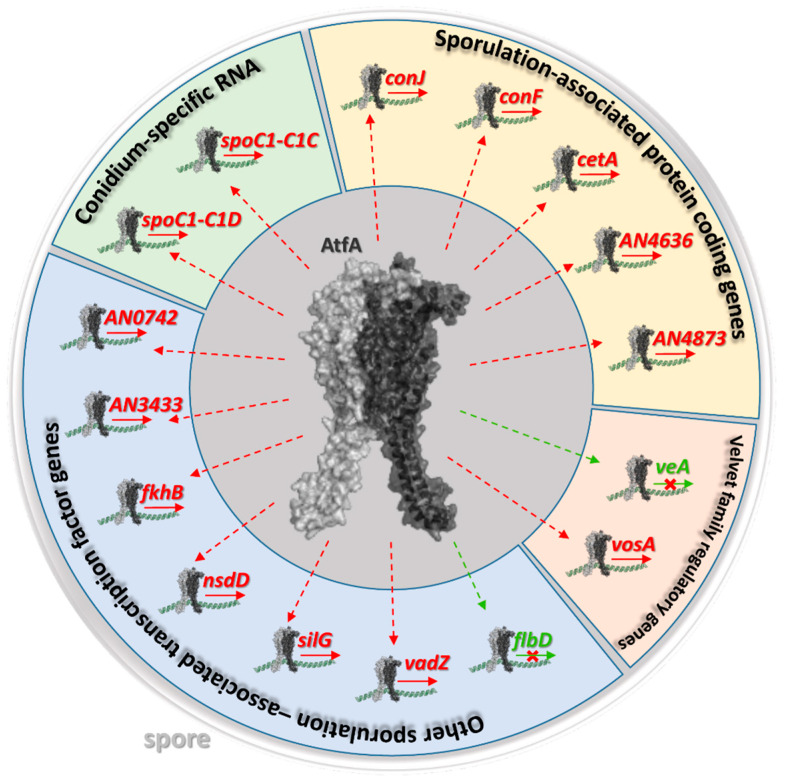
**A major fraction of genes involved in the conidiogenesis of *Aspergillus nidulans* and whose expression is influenced by AtfA binding in their promoters**. For functional characterization of the genes presented here, see Table S3, Sheet 6, the annotation based on data available in the FungiDB database (FungiDB-68_AnidulansFGSCA4.gff), the results of GO enrichment analysis in shown Table S4, and/or the following sections. The thin arrows above the genes indicate their expression. The thick arrows represent the effects of the genes, showing the processes they influence.

**Figure 5:**
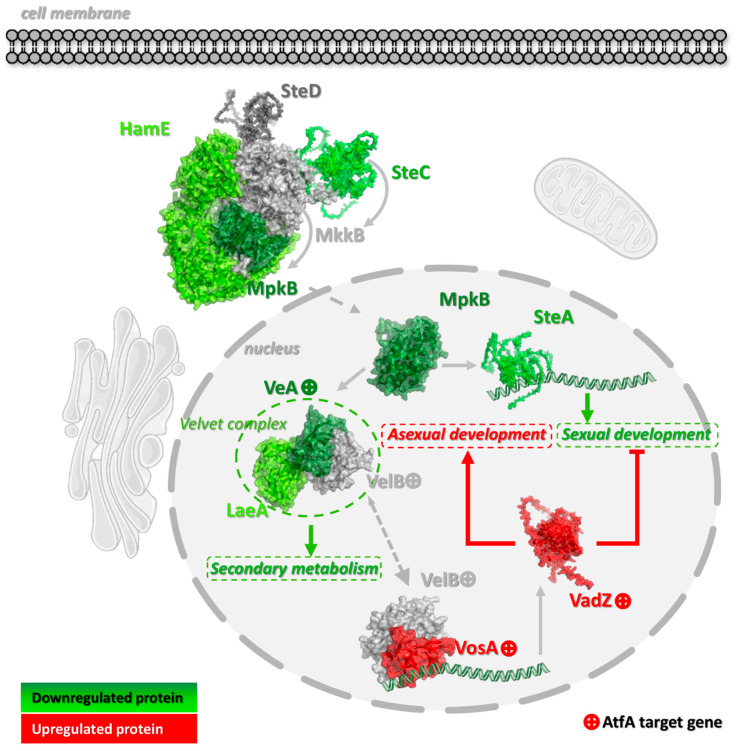
**Expression changes in sporulation-related genes in response to the AtfA transcription factor recorded in *A. nidulans* conidia.** Red indicates increased expression, green indicates decreased expression, and gray indicates no change in gene expression. Components marked with a “+” symbol are those with an identified ChIP-seq peak. Protein structures were visualized using AlphaFold3 predictions (Table S5). Solid arrows represent regulatory effects, while dashed arrows indicate transports.

**Figure 6:**
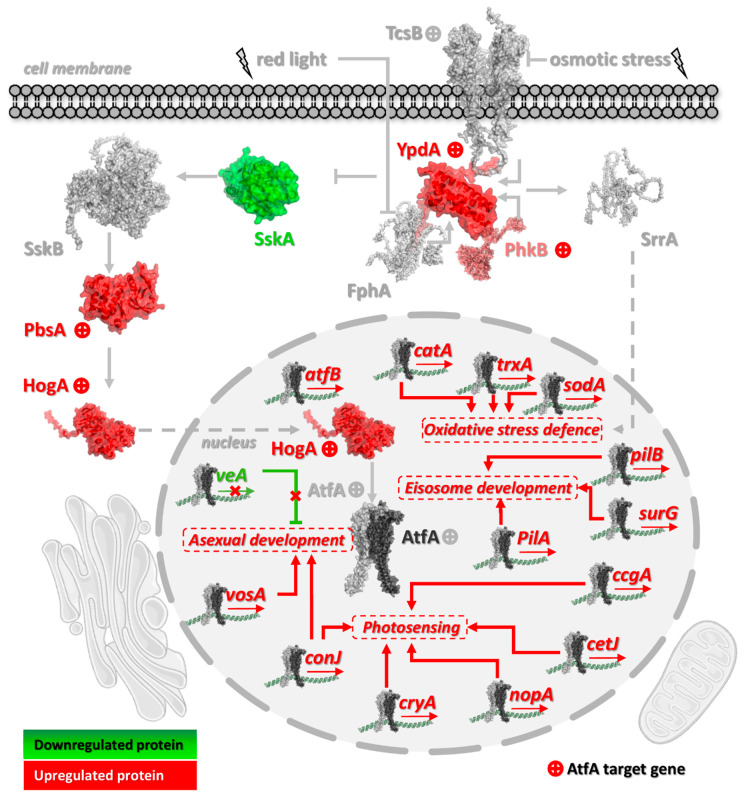
**Expression changes in the HogA pathway components in response to the AtfA transcription factor found in *A. nidulans* conidia.** Red indicates increased expression, green indicates decreased expression, and gray indicates no change. Components marked with a “+” symbol are those with an identified ChIP-seq peak. Protein structures were visualized using AlphaFold3 predictions (Table S5). Solid arrows represent regulatory effects, while dashed arrows indicate nuclear transport. The lightning symbol represents the external environmental signal (two types: red light and osmotic stress). In the nucleus, predicted target genes of homodimeric AtfA are shown (only a subset of key targets is displayed).

**Figure 7:**
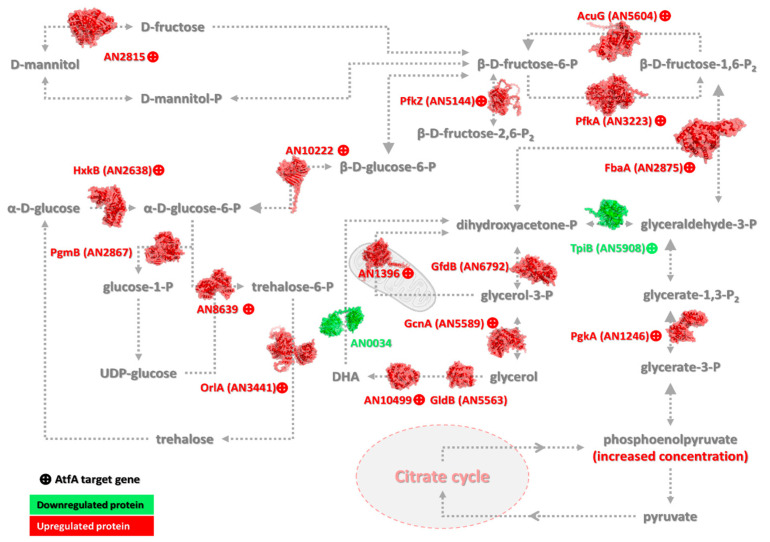
**Expression changes in central carbon metabolism pathways in response to AtfA.** The conversion and movement of precursors and intermediates, as well as the direction of the associated processes, are indicated by dashed arrows. Proteins catalyzing the respective reactions were visualized using AlphaFold3 predictions (Table S5). Only proteins showing expression changes are included in the schematic, where red indicates increased expression and green indicates decreased expression. A “+” symbol denotes genes with a ChIP-seq peak identified in their promoter region.

**Figure 8:**
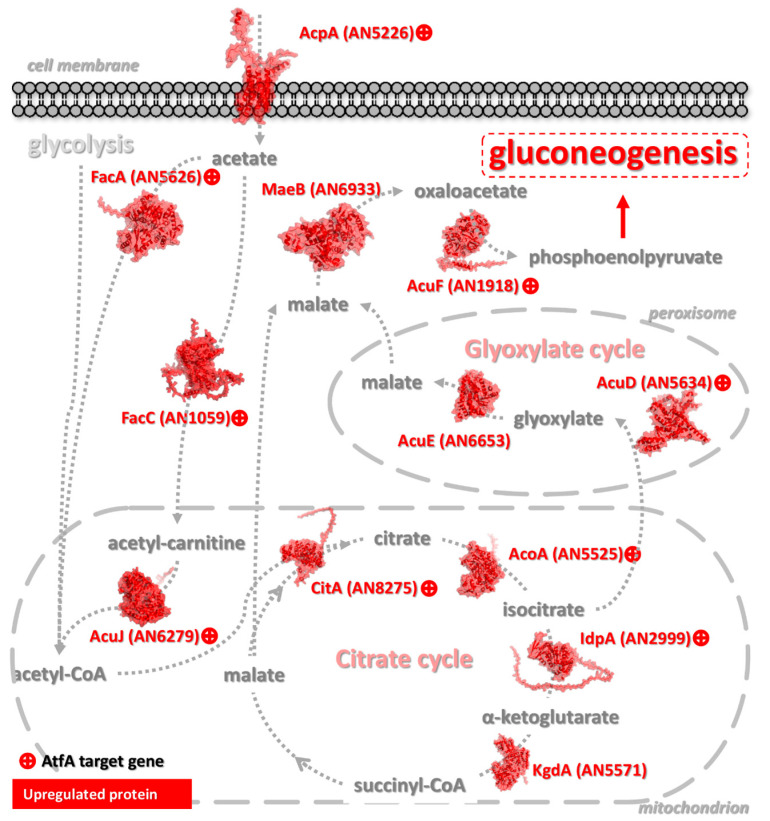
**Changes in the central primary metabolic pathways TCA and glyoxylate cycles and acetate utilization in response to the AtfA transcription factor**. The conversion and movement of precursors and intermediates, as well as the direction of the associated processes, are indicated by dashed arrows. Proteins catalyzing the respective reactions were visualized using AlphaFold3 predictions (Table S5). Only proteins showing expression changes are included in the schematic, where red indicates increased expression. A “+” symbol denotes genes with a ChIP-seq peak identified in their promoter region.

In the following sections, Figure 4, Figure 5, Figure 6, Figure 7 and Figure 8 display representative gene groups selected based on annotation data available in the FungiDB database (FungiDB-68_AnidulansFGSCA4.gff), direct manual literature search (Table S3, Sheet 6), and the results of GO enrichment analysis (Table S4, Figure S4).

AtfA binding sites were assessed in both the TSS–500 region and the broader −1000 to +50 bp window around the TSS [46]. Unless otherwise indicated, these genes possessed AtfA-bound promoters showing altered expression in Δ*atfA* compared with wild-type in one of the regions shown in Figure 2 in both unstressed and MSB-exposed cultures. Genes whose activity is affected by AtfA-binding in their promoters only in untreated (Untr. only) or MSB-treated (MSB Tr. only) cultures are indicated in parentheses. The complete sets of “Untr. only” and “MSB Tr. only” genes can be found in Table S3, Sheets 1–6, where these genes are highlighted in different colors.

#### 3.3.1. AtfA Is Involved in the Regulation of Conidiogenesis—A General Overview

Our study provides genome-wide mapping of AtfA binding in the model filamentous fungus *A. nidulans* and defines how this key transcription factor coordinates asexual sporulation with primary metabolism and stress preparedness. As we have shown in previous sections, we identified the conserved CRE-type AtfA motif and showed that AtfA binding is largely stress-independent, revealing a pre-emptive regulatory architecture that links metabolic and developmental programs. Such constitutive AtfA occupancy represents a biologically meaningful mechanism by which conidia remain “pre-armed” for rapid activation of stress-response and metabolic genes. This anticipatory regulation allows for immediate adaptation to a rapidly changing environment without requiring new transcription-factor recruitment. Furthermore, it should be kept in mind that molecular oxygen itself has a significant impact on aerobic microorganisms, including fungi. O2 and its toxic derivatives (ROS), mainly produced by mitochondrial respiration and some peroxide-forming enzymes, constantly challenge cells, forcing them to adapt or isolate themselves through diverse forms of cell differentiation, such as sexual and asexual sporulation [9,10]. It is therefore reasonable to assume that conidiogenesis, primary metabolism, and defense against environmental stress, especially oxidative stress, are largely synchronized and even coregulated at the transcriptional level in fungal cells undergoing asexual development, and that coregulation at this level may favor constitutively active, “universal” transcription factors such as AtfA.

Some of the genes targeted by AtfA are directly related to conidiogenesis or are involved in the initiation and regulation of asexual development (Figure 4 and Figure S4, Tables S3 and S4). Furthermore, ChIP-seq data combined with global gene expression changes [5] clearly show that AtfA is directly involved in the co-regulation of MAPK signaling, eisosome assembly, antioxidant defense, expression of light-responsive transcription factors, and biosynthesis of stored carbohydrates—several important elements of the complex network of cellular processes intrinsically linked to and ensuring the formation of asexual spores [3], which we will discuss in detail in the following chapters. We demonstrate that such a combination of available experimental data helps us distinguish direct transcriptional control from indirect downstream effects, significantly expands our knowledge beyond previous transcriptomic studies, and will define AtfA as one of the central transcriptional regulators of fungal biology. Importantly, integrating ChIP-seq with RNA-seq data allows us to connect AtfA’s promoter occupancy with specific gene networks and phenotypes.

Although it is reasonable to propose that AtfA serves as a master integrator of developmental and stress-related processes, the upstream cues and signaling pathways that enable constitutive promoter binding or activate promoter-bound AtfA during development, dormancy, and germination are still unclear. This remains an open question and should be kept in mind when reading the subsequent sections.

#### 3.3.2. AtfA Modulates the Expression of Other Transcription Factors

As shown in Figure 4, the versatile regulatory functions of AtfA in the initiation and maintenance of asexual sporulation may be manifested through the binding of AtfA to genes encoding other transcription factors organically linked to sporulation processes. In general, the signaling pathways operating in hyphae and preparing vegetative tissues for asexual development are sequentially deactivated, with a concomitant reorganization of the regulatory network, maintaining the integrity of hyphae, newly emerging structures bearing conidia, and the conidia themselves [1,2,3].

As previous global gene expression data indicated [5], three genes encoding the components of the SteC-MkkB-MpkB-SteD-HamE pheromone module regulating stress responses, secondary metabolism, and asexual development in *Aspergillus* colonies [50,51], the *mpkB* and *steC* protein kinase and *hamE* scaffold protein genes, are under negative AtfA control in the wt strain, together with the *velvet* regulator *veA*, the sexual transcription factor *steA*, and the methyltransferase-domain protein *laeA* genes (Figure 5). Importantly, only *veA* possesses an AtfA-bound promoter showing altered expression in Δ*atfA* compared with wild-type (Figure 4 and Figure 5), and *flbD* coding for an early, c-Myb transcriptional activator of asexual development [52,53] was also negatively affected (Unstr. only) by AtfA (Figure 4). Because AtfA is typically a positive transcriptional regulator (Figure 3), its effect on *veA* and *flbD* expression may be overcompensated by other not-yet-identified transcriptional regulators.

All these transcriptional changes point towards the general downregulation of asexual and sexual developments and sterigmatocystin production [2,54,55,56]. Under these conditions, the *vosA velvet* protein gene (Untr. only) with an AtfA-bound promoter may contribute to the maintenance of asexual development during the final stage of conidiogenesis [2,3]. VosA, together with VelB, which also contains an AtfA binding site without any effect on its expression (Figure 5, Table S3, Sheet 1), play a key role in conidiospore maturation (including trehalose biogenesis) and dormancy [2,3,57,58,59]. Furthermore, VosA-VelB-mediated activation of the Zn(II)_2_Cys_6_ transcription factor gene *vadZ* (Figure 4 and Figure 5), whose expression is also affected by AtfA, may also be involved in the regulation of asexual spore formation and maturation. Previous studies demonstrated that VadZ initiates asexual development via the activation of the BrlA-AbaA-WetA central regulatory pathway of sporulation and may also suppress sexual development and sterigmatocystin production [3,60].

AtfA may also support conidiogenesis through the forkhead transcription factor gene *fkhB* [61,62], which has an AtfA-bound promoter (Figure 4), because the deletion of *fkhB* negatively affected both the heat stress tolerance and the trehalose content of conidia in an earlier study [61]. Interestingly, the gene encoding the negative conidiogenesis transcriptional regulator NsdD (Unstr. only), which directly interferes with the expression of the central sporulation regulatory gene *brlA* [63] and *silG* coding for a negative regulator of sexual development in light [64,65], was also among the AtfA-target genes (Figure 4). The apparent negative regulatory effects of AtfA on the expression of some genes with AtfA-bound promoters (Figure 4, Table S3, Sheet 6) are likely mediated through AtfA-controlled repressors (e.g., VosA, NsdD or SilG).

Consistent with the positive role of AtfA in regulating conidiation, Kocsis et al. (2022) [26] reported a significant reduction in conidiospore yield in surface Δ*atfA* cultures, but the same strain also showed a defect in cleistothecium formation. These observations reflect the remarkable complexity of the interplay of regulatory elements that balance and fine-tune sexual and asexual development and, hopefully, will initiate further research in this area.

#### 3.3.3. HogA/SakA MAPK Signaling and Other Protein Kinases

The SskB (MAPKKK) → PbsB (MAPKK) → HogA/SakA (MAPK) → AtfA stress signaling and regulatory pathway (Figure 6) is crucial for proper and long-term environmental stress protection against osmotic, oxidative, cell wall integrity-damaging, and thermal stresses, not only in vegetative tissues but in resting conidia as well [3]. It is noteworthy that two pivotal elements of this key pathway operate under AtfA control, namely PbsB [66,67] and HogA/SakA [25,67,68,69,70]. The loss of positive control of the MAPK pathway by AtfA in Δ*atfA* cultures explains well the increased oxidative stress sensitivity of mycelia and the reduced heat and oxidative stress tolerance of mutant conidia [23,24,25,71]. Not surprisingly, prolonged storage at 4 °C also decrease the vitality of the Δ*atfA* gene deletion mutant strain [23,24].

YpdA, an ortholog of the *Saccharomyces cerevisiae* histidine-containing phospho-transfer protein Ypd1, receives a signal from the budding yeast Sln1 osmosensor ortholog TcsB histidine kinase and forwards it to SskA (budding yeast ortholog is Ssk1) and SrrA (*S. cerevisiae* ortholog is Skn7) response regulators (Figure 6) [66,72]. In *S. cerevisiae*, inactivation of Ssk1 by Ypd1-dependent phosphorylation is required for activation of the HOG pathway, which occurs under high osmolarity stress conditions [73]. Ypd1-YpdA-dependent stress sensing and signaling can mediate a variety of environmental stress (including oxidative stress) signals in addition to osmotic stress [74,75,76]. Decreasing or eliminating the activity of YpdA by either genetic manipulation or antimycotics like fludioxonil can drive fungal cells to death due to the improper activation of the HOG pathway [66,72,77,78]. Therefore, the AtfA-dependent positive regulation of *ypdA* can present negative feedback on the HOG pathway similar to budding yeast, where Ypd1 has a stress response buffering effect, hence, its pool being typically large in vegetative cells [79]. Since the expression of some genes with AtfA-bound promoters is apparently negatively regulated by AtfA (Figure 4 and Figure 6, Table S3, Sheet 6), we can hypothesize that ultimately feedback interactions within the MAPK signaling cascade may also lead to downregulation of some AtfA-targeted genes.

Importantly, AtfA itself operates downstream of HogA/SakA MAPK [25,70,80], and its gene also possesses an AtfA binding site which is indicative of the likely autoregulation of *atfA* gene expression. Similarly, an Atf1 binding site is also present in the promoter of *S. pombe atf1* [19,20,21,22], and a positive feedback loop may accelerate the response of *atf1* to H_2_O_2_ stress [19]. Although self-regulation of *A. nidulans* AtfA through an autoregulatory feedback loop also seems reasonable, experimental elimination/modification of the potential AtfA binding site is necessary to verify this hypothesis.

Just like the Atf1-Pcr1 bZIP transcription factor pair, which regulates many physiological processes, including oxidative stress response, in fission yeast [19,22,81] the bZIP transcription factor AtfB may support AtfA in its regulatory roles in *A. nidulans* [5,26]. It is noteworthy that the Δ*atfB* strain had a NaCl hyperosmotic stress-sensitive phenotype, and mutant conidia were also heat sensitive [26]; furthermore, the expression of *atfB* itself was also AtfA-dependent (in both mycelium and conidia) and responded to MSB exposure (in mycelium) in *A. nidulans* [5]. Similarly to fission yeast, where Atf1 binds to the promoter of its dimerization partner gene pcr1 [22], *A. nidulans* AtfB harbors an AtfA-bound promoter showing altered expression in Δ*atfA* compared with wild-type in conidia harvested from either unstressed or MSB-treated surface agar cultures.

Interestingly, a set of histidine kinase genes including *phkB*, *hk2*, *hk-8-2*, *hk-8-3*, and *hk-8-7* were uniformly under AtfA control (*hk-8-7* MSB Tr. only; Table 3), which is in good accordance with previous observations [5,28,29]. These histidine-containing phosphotransfer proteins take part in complex regulatory processes in filamentous fungi including the orchestration of asexual and sexual developments in addition to stress responses [82]. PhkB, a homolog of *S. pombe* Mak1 phosphorelay sensor kinase, is phosphorylated in the presence of H_2_O_2_ in *A. nidulans* [83] and has been linked to the regulation of conidiation in *A. fumigatus* [84].

Another important observation is the AtfA-dependent positive control of the *pkaA* protein kinase A gene that supports both conidiospore germination and vegetative growth in *A. nidulans* [3,56,85,86]. At first glance, this regulation seems to be futile under conidiogenesis, but this kinase will be crucial later under germination including proper stress defense [85,87,88]. To resolve this paradox, the *pkaR* regulatory subunit gene negatively regulating PkaA [3,89] is also under positive control by AtfA, preventing premature activation of PkaA. Other protein kinases under AtfA control include SrkA/CmkD, a physical interacting partner of HogA/SakA MAPK [70], and Isr1 (Untr. only). Isr1 is targeted by the conidium development transcriptional regulator WetA [88] and Nnk1 protein kinase (AtfA-controlled, Untr. only), which is phosphorylated by MpkA and SakA MAPKs under caspofungin exposures in *A. fumigatus* [90]. These observations clearly suggest that the versatile physiological roles of AtfA may be based, at least in part, on the modulation of the expression of some important protein kinase genes [5,28].

Protein phosphatases are of paramount importance in regulating important cellular processes like mitosis [91] and are essential to various signal transduction pathways [92]. Not surprisingly, AtfA also contributes to the fine-tuning of these processes and pathways via binding to the promoters of important protein phosphatase genes including *ptcD* (Untr. only) and *ptcE* [93] and *ptpA* (MSB Tr. only), whose protein product physically interacts with SakA [83].

**Table 3:** Histidine kinase-encoding genes possess an AtfA-bound promoter showing altered expression in Δ*atfA* compared with wild-type.

Gene ID	Gene Name	Functional Description	References	AtfA and AtfB Dependent Regulations (Kocsis et al., 2023) [5]	
				Unstressed Culture ^‡^	MSB-Exposed Culture ^‡^
AN3101	*phkB*	Putative histidine-containing phosphotransfer protein	Suzuki et al., 2008; Hagiwara et al., 2007 [82,94]	**AA**	**AA**
AN7945	*hk2*	Putative histidine-containing phosphotransfer protein	Suzuki et al., 2008; Hagiwara et al., 2007; Azuma et al., 2007; Bahn, 2008 [82,94,95,96]	**AA**	**AA**
AN4113	*hk-8-2*	Histidine kinase, histidine-containing phosphotransfer protein	Suzuki et al., 2008; Hagiwara et al., 2007; Azuma et al., 2007; Bahn, 2008 [82,94,95,96]	**AA**	**AA**
AN6820	*hk-8-3*	Putative histidine-containing phosphotransfer protein	Suzuki et al., 2008; Hagiwara et al., 2007; Azuma et al., 2007; Bahn, 2008 [82,94,95,96]	**AA**	**AA**
AN9048	*hk-8-7*	Orthologue of *S. cerevisiae GCN20*	Suzuki et al., 2008; Hagiwara et al., 2007; Azuma et al., 2007; Bahn, 2008 [82,94,95,96]		

^‡^—AA: gene regulated putatively by AtfA but not by AtfB, based on transcriptomics data (Kocsis et al., 2023) [5].

#### 3.3.4. Light-Dependent Regulation of Conidiogenesis

Light is one of the most important environmental factors stimulating conidiogenesis [2,97,98]. In the central regulatory pathway of sporulation [1], BrlA-elicited conidiation is clearly based on FphA, a red-light sensing phytochrome [99]. The FphA photoreceptor physically interacts with the YpdA phosphor-transmitter, which leads to the activation of the HogA/SakA MAPK pathway [100], the subsequent phosphorylation of AtfA, and the blockage of the germination of conidiospores [25,100]. Similarly to *ypdA*, the red-light upregulated AN5401 gene also harbored an AtfA binding site (Table S3), but its regulation was FphA-independent [101]. Two light-inducible genes frequently used in light-dependent regulation studies, *ccgA* and *conJ* [Figure 4 and Figure 6; [100,102,103]], were under AtfA control [96], which is in line with our transcriptomics and ChIP-seq data (Table 4). A large group of other light-responsive genes was also subject to AtfA control in both unstressed and MSB-exposed *A. nidulans* mycelia and conidia [5], and ChIP-seq data confirmed direct AtfA control of the majority of these genes in conidia (Table 4). Importantly, other light-responsive genes like *nopA* (encoding a fungal opsin [99,104]), *cryA* (coding for a deoxyribodipyrimidine photo-lyase [100]), an *S. pombe uve1* DNA repair endonuclease gene homolog (AN0604), *cetJ* (stands for “conidia enriched transcript J” [44,99], and some other putative clock-control protein genes (loci AN4299 and AN5056) were under AtfA control. All the observations underline the outstanding importance of AtfA in the involvement of light-dependent processes in fungal asexual development, which progressed in both unstressed and MSB-treated cultures.

#### 3.3.5. Formation and Maintenance of Subcellular Conidial Organelles and Structures

Important genes that are key players in the formation and functioning of subcellular conidial organelles and structures including mitochondria, endoplasmic reticulum (ER), eisosomes, vacuoles, proteasomes, ribosomes and cytoskeleton elements harbored AtfA binding sites in their promoters (Figure S2).

In *A. nidulans* conidiospores, the expression of a number of mitochondrial proteins was under direct transcriptional control by AtfA, hence contributing to the formation and preservation of the integrity and function of this important cell organelle [112,113,114]. For example, AtfA bound to the promoters of the *S. cerevisiae YME2* ortholog AN10634 gene with a putative role in mitochondrial biogenesis machinery [115], the respiratory *cycA* cytochrome c gene [116,117], *mcr1* coding for mitochondrial NADH-cytochrome b5 reductase [118] (Untr. only), whose baker’s yeast’s ortholog *MCR1* has antioxidant functions [119], *oliC* encoding Subunit 9 of the mitochondrial inner membrane F1F0-ATPase complex [120], and *dicB*, a putative dicarboxylate-tricarboxylate carrier gene [121] (MSB Tr. only). Further, AtfA-target genes include the *mdm34* ERMES (Endoplasmic Reticulum and Mitochondria Encounter Structures) gene, stabilizing organelle shape and mtDNA [122], and *aifA* Apoptosis-Inducing Factor (AIF)-like mitochondrial oxidoreductase gene, an important element of oxidative and farnesol stress defense [123].

Other cell organelles with AtfA-target genes included ER (at Yop1 ER membrane-bending protein, contributing to ER inheritance and involved in response to tunicamycin—an *N*-glycosylation inhibitor—which elicited ER stress [124,125]), vacuoles (at VcxA vacuolar H^+^/Ca^2+^ exchanger [126,127]), and autophagosomes (at Atg12 ubiquitin-like protein [128]; Untr. only). Autophagy in resting conidia may help these spores to safeguard their longevity and vitality [129,130] and may also support these propagules when they germinate under nitrogen-limited environments [131].

In addition to vacuoles, AtfA also affected ubiquitination/proteasomal protein degradation via regulating the expression of F-box protein genes {AN3203 (MSB Tr. only), AN5209, AN6183, AN8051 [132]; Table S3}. Interestingly, expression of the AtfA-controlled HECT-type ubiquitin ligase-interacting protein gene *apyA* [133] increased in unstressed cultures of the Δ*atfA* strain. In the nuclei, AN10461 Ulp1 family protease [134] (Table S3) is likely to take part in SUMO deconjugation processes [135], and Ulp1 is sequestered into the nuclei of *S. cerevisiae* upon stress, allowing for an increase in steady-state SUMO conjugation levels in yeast cells [136].

One of the most interesting and unexpected findings was the clearcut AtfA-dependency of the regulation of an important and well-characterized group of eisosomal proteins including PilA, PilB, SurG, and Nce102 [137,138,139]. Eisosomes are subcellular organelles involved in plasma membrane organization, regulation of plasma membrane lipid homeostasis, cell wall biosynthesis, and protection of fungal cells from environmental (including oxidative) stress, and they may play an indirect role in endocytosis [140,141,142,143]. The eisosomal proteins PilA, PilB, and SurG form tightly packed punctate structures only in the cell cortex under maturation of *A. nidulans* conidia, which disassemble during germination [137]. Another protein, Nce102, stabilizes PilA/SurG foci in the head of germlings; meanwhile, PilA and Nce102 negatively regulate sphingolipid biosynthesis and contribute to H_2_O_2_ and paraquat (but not menadione) oxidative stress defense in *A. nidulans* [139]. The AtfA-control of another gene *slmA* (putatively encodes a phosphatidylinositol-4,5-bisphosphate binding protein [144]) localized to the eisosomes indicates that AtfA may also strengthen actin cytoskeleton organization under environmental stress like the orthologous Slm1/2 proteins in baker’s yeast [145,146]. Further studies are needed to clarify if other AtfA-targeted genes likely taking part in the metabolism of phosphatidylinositol phosphates {AN0664—*plcA* (MSB Tr. only), AN2877, AN6367} are also working together with eisosomal proteins to regulate the homeostasis of plasma membrane lipids [147].

Considering cytoskeletal elements, *fimA*-encoding fimbrin (an actin-binding protein) was under AtfA control, which underlines the role of AtfA in the preparation of resting conidia to break dormancy and germinate [148,149]. FimA functions include proper polarity establishment during germination of conidia as well as maintenance of hyphal growth via endocytotic recycling of polarity markers through subapical regions to the hyphal apices [148]. The AN3022 gene coding for β-tubulin folding cofactor C (Table S3, Sheet 1) and required for proper folding and polymerization of this microtubule-building protein [150] also possesses an AtfA-bound promoter. The expression of the *uncB* kinesin-3 motor protein gene [151,152] (Untr. only) was also controlled by AtfA in unstressed cultures.

#### 3.3.6. Involvement of AtfA in the Control of Transcription and Translation Machineries

General control of transcription by AtfA was merely characteristic under MSB stress via *tbp* (MSB Tr. only), the putative TATA-box binding protein gene [153]. Interestingly, RNA processing, ribosome biogenesis, and translation were relatively slightly under AtfA-dependent regulation, but *ubi1* ubiquitin-large ribosomal subunit fusion protein gene [154,155] (Untr. only; heat-shock and H_2_O_2_-inducible), *ppe1* encoding a small subunit mitochondrial ribosomal protein with carboxyl methyl esterase activity in *S. cerevisiae* [156,157], *helA* coding for a ATP-dependent DEAD-box RNA helicase [158,159], and AN6700 putative translation elongation factor eEF-3 gene [160,161,162] (Table S3, Sheet 1) possessed AtfA-bound promoters showing altered expression in Δ*atfA* compared with wild-type. Importantly, the expression of the AN10614 gene, a *S. cereviae STM1* ortholog, was also under AtfA control. Baker’s yeast Stm1 ribosome-associated protein is needed to increase the number of ribosomes under nitrogen starvation [163,164]. In *A. nidulans*, the Stm1 ortholog is a DenA deneddylase 1 interacting protein and, hence, may modulate developmental programs via this interaction [165].

Fine-tuning of conidiation by AtfA may also rely on the expression of histone H4.1 (Untr. only), which is abundant in *A. fumigatus* conidial proteome [166] as well as AN7208 SET domain protein [167] (a SET domain typically has methyltransferase activity) and PhoW, a putative chromatin modification-related protein [168] (Untr. only).

#### 3.3.7. Preservation of Resting Conidia

By combining available transcriptomic and ChIP-seq data, we concluded that AtfA is the main integrator linking the regulation of primary metabolism (biosynthesis of important reserve carbohydrates, amino acids, glutathione, etc.) as well as the expression of environmental stress response proteins during conidiogenesis with developmental progression.

For example, trehalose and polyols (mannitol, erythritol and glycerol) are essential products of primary metabolism, so-called compatible solute molecules, which are required for long-term stability and survival of resting conidia [27,44,57,169,170,171,172,173,174,175,176]. Supplying dormant conidia with appropriate carbon (trehalose, alditols) and nitrogen (amino acids, e.g., glutamate) reserves and mobilizing these compounds at the right time during germination are of pivotal importance for successful propagation [5,27,169]. Mature *A. nidulans* conidia contain predominantly trehalose and mannitol as carbon reserves, and glycerol temporarily accumulates during germination [45] (Table 1).

Considering trehalose metabolism, AtfA controls OrlA trehalose 6-phosphate phosphatase (Figure 7), which plays versatile roles in the stabilization of conidiospores, especially at an elevated temperature, because the gene disruptant mutant spores are chitin deficient and, hence, highly unstable [177]. Similar osmoremediable phenotypes were described for the Δ*orlA* mutant of the opportunistic human pathogen *A. fumigatus*, which was avirulent [178]. Another AtfA-targeted trehalose biosynthetic gene encodes AN8639 α,α-trehalose-phosphate synthase (UDP-forming) [5] (Figure 7). In mannitol metabolism, two genes involved in mannose metabolism, including AN2815 mannitol 2-dehydrogenase [179,180] (catalyzing D-mannitol ⇌ D-fructose conversion; Figure 7) and AN1715 mannose-6-phosphate isomerase (interconverting fructose 6-phosphate and mannose-6-phosphate [181,182]), possessed AtfA binding sites in their promoters.

Genes coding for AN8010 (Untr. only; *gsyA*) glycogen synthase (present in hyphal tips and sub-apical cells [183]) and AN10060 glycogen debranching enzyme (a homolog of *S. cerevisiae GBD1* bifunctional α-1,4-glucanotransferase and α-1,6-glucosidase [184]) also possess AtfA binding sites in their promoters and may be related to conidial germination.

Maturing conidia also store various amino acids, among which the accumulation of glutamate was AtfA-dependent in *A. oryzae* [27]. As expected, some amino acid metabolic genes were also found to be regulated by AtfA in this study. Not surprisingly, AtfA significantly affected glutamate/glutamine metabolism at GltA glutamate synthase (NADH) [185] (Untr. only), AN4901 glutaminase [186], and AN3829 succinate-semialdehyde dehydrogenase sites [NAD(P)^+^] [81,186,187]. The latter enzyme is likely to contribute to the maintenance of the redox milieu of oxidative stress exposed cells via its NADH/NADPH production [188]. Glycine, serine, and threonine metabolism may also be modulated by AtfA at *codA* choline oxidase gene under asexual sporulation [161] (Untr. only). CodA may be required in the biosynthesis of the osmoprotectant glycine betaine under menadione exposure [189].

Important metabolic enzymes like xanthine oxidase and nitrate reductase incorporate a molybdenum-containing cofactor, molybdopterin [190]. AtfA also controls molybdopterin cofactor-related genes, including AN9038 molybdopterin biosynthetic protein [191] and AN0183 molybdopterin binding domain protein, with increased quantity in benzoate-exposed *A. nidulans* mycelium [192] and with decreased quantity in conidia after 60 min germination [193].

Glutathione/glutaredoxin and thioredoxin systems are crucially important in maintaining the redox homeostasis in fungal cells [194,195,196], and the level of glutathione reduced form decreased in germinating *A. flavus* conidiospores but increased later [197]. In *A. oryzae*, the glutathione system was likely dysfunctional in the Δ*atfA* mutant, and the AO080539000104 thioredoxin gene was under AtfA control in conidia [27]. In *A. nidulans*, only the *trxA* thioredoxin gene [198] was controlled by AtfA in unstressed *A. nidulans* cultures. Nevertheless, considering sulfur-containing amino acid metabolism, AN6105 and AN11039 carbon-sulfur lyases were under AtfA control, and the expression of AN11039 lyase was light-regulated and FphA phytochrome-dependent [103]. The promoter of the gene AN6963 coding for sulfhydryl oxidase, a paralogue of the *A. fumigatus* gliotoxin neutralizing enzyme GliT, was also bound by AtfA [199]. AN10197 encodes an AtfA-controlled putative sulfonate biosynthesis enzyme, and the quantity of its *A. fumigatus* ortholog AFUA_8G04550 decreased in germinating conidia [200].

In addition to TrxA thioredoxin, AtfA positively regulates a number of well-characterized other oxidative stress defense protein genes encoding the major conidium-specific catalase CatA [5,25,201] and the Cu/Zn superoxide dismutase SodA [202] (Untr. only). Furthermore, *crdA* encoding a metallothionein-like protein (probably involved in heavy metal stress defense [203]) and *dlpA* coding for a dehydrin-like protein (effective against various types of environmental stress [167]) were also under AtfA control. These findings are in line with the prominent role of AtfA in the orchestration of environmental stress defense, especially concerning combating oxidative stress in *A. nidulans* [4,15,23,24,29]. Unexpectedly, loss of *atfA* led to the significant, paradoxical upregulation of *pyroA* coding for an important pyridoxine biosynthetic protein [204]. Pyridoxine (vitamin B_6_) is an efficient antioxidant [203] which protects against photosensitizers [205] and *tert*-butyl hydroperoxide (*t*BOOH)-elicited oxidative stress [24]. Upregulation of the vitamin B_6_ biosynthetic pathway by the yet-to-be-identified regulatory mechanism may help Δ*atfA* mutant conidia compensate for the lack of upregulation of AtfA-dependent elements of the oxidative stress defense system, including other antioxidant biosynthetic pathways.

AtfA also regulated directly a wide spectrum of transmembrane transporters contributing to the maintenance of cellular homeostasis. For example, EnaA putative potassium-transporting ATPase (MSB Tr. only) plays a crucial role in the adaptation to alkaline pH [206] and sodium ion detoxification [127]. Other transporter genes with AtfA-bound promoters included AN2822 aquaporin [207,208] as well as AN7138 (Untr. only), AN7295, and AN8122 Major Facilitator Superfamily membrane transport protein genes [134,186,208,209]. The AN12035 putative carbonate dehydratase contributing to the maintenance of proper intracellular acid–base balance (a homolog of *A. fumigatus* Afu4g09420 putative carbonic anhydrase [210]) and also harbored an AtfA-bound promoter showing altered expression in Δ*atfA* compared with wild-type.

Some conidium-specific genes, whose physiological functions have not yet been well characterized, are also under the control of AtfA. For example, AtfA positively regulates two conidium-specific protein genes *conF* and *conJ* (Figure 4 and Figure 6), which negatively affected glycerol or erythritol levels and desiccation tolerance of conidia [44], two conidium-specific RNA genes, *spocC1-C1C* and *spocC1-C1D*, as well as *cetA* coding for a secreted thaumatin-like protein, playing an essential role in early conidial germination [211] (Figure 4). Interestingly, *awh11* encoding a conidium expressed protein with homology to small heat-shock proteins [211] was upregulated in MSB-exposed Δ*atfA* mutant cultures. *awh11* expression was also derepressed in Δ*stuA* mutants [210], where StuA is an APSES family transcription factor developmental modifier regulating proper conidiospore formation [1,3,212].

AtfA directly controls important environmental stress defense genes as demonstrated by ChIP-seq data, but the total number of these genes is relatively low, approximately 13.7% of those collected in the *Aspergillus nidulans* Stress Protein Database updated version 2024 (46 from 342 genes; Tables S1 and S3, Sheet 1). Furthermore, the number of stress-protective genes recognized by AtfA only during MSB exposure is very small, only 4; in fact, half of those bound by AtfA are only in stress-free cultures (seven genes).

The number of AtfA-regulated stress response genes with demonstrated physiological functions in conidiation, maintenance of resting conidia, and germination is also relatively low, reaching 19 (5.6% of total; Tables S1 and S3). These data confirm that, similar to *S. pombe* Atf1 [22], *A. nidulans* AtfA binds to the vast majority of target gene promoters, even stress defense genes, regardless of the presence or absence of oxidative stress. Upregulation of constitutive stress protection genes by AtfA provides adequate stress protection to spores during conidiogenesis and makes further adaptation to MSB-induced oxidative stress unnecessary [5]. Furthermore, the large number of genes under AtfA control but with no obvious role in stress defense suggests that the primary function of AtfA lies elsewhere in the precise spatial and temporal coordination of conidiogenesis and the primary metabolism that supports it.

#### 3.3.8. Fueling Biosynthetic Processes Progressing in Conidia

High-density asexual sporulation requires a huge supply of nutrients including glucose [42]. As a consequence, starting glucose was completely taken up and metabolized in unstressed cultures at 2 d incubation; meanwhile, glucose utilization and conidiogenesis were slower under MSB stress but reached wild-type levels at 3 d incubation (Table 1). In a previous transcriptomics-based study by Kocsis et al. (2023) [5], it was demonstrated that many important elements of carbohydrate metabolism were under AtfA-dependent control in *A. nidulans* conidia harvested from either unstressed control or MSB-exposed cultures. Significant enrichment of primary metabolic pathway genes was detected in glycolysis and gluconeogenesis, pentose phosphate pathway, fructose and mannose metabolism, pyruvate metabolism, glyoxylate and dicarboxylate metabolism, TCA cycle, and the Leloir pathway [5]. All these primary metabolic pathways are needed to synthesize the building blocks (e.g., cell wall biopolymers) and reserves (e.g., trehalose and alditols) necessary for conidiogenesis and spore survival.

Considering carbohydrate uptake, the sugar transporters MstB [213] and AN4277 [77] as well as AN11016 fludioxonil-responsive putative malate transporter [77,190] were positively regulated by AtfA. The glycolytic pathway converting glucose to pyruvate is positively regulated by AtfA at some key enzymes including HxkB atypical hexokinase-like protein [214,215], glucose-6-phosphate 1-epimerase [216,217], PfkA 6-phosphofructokinase [218], FbaA fructose-bisphosphate aldolase [219], PgkA phosphoglycerate kinase [215,220], and TpiB triose-phosphate isomerase [153] (Figure 7). The alternate glucose metabolic pathway parallel to glycolysis, the pentose phosphate pathway, which supplies cells with reducing power (NADPH) and ribose, was under AtfA control at AN0285 6-phosphogluconolactonase and AN5907 ribose-5-phosphate isomerase [186].

Gluconeogenesis can be crucially important when a high amount of glucose is incorporated into cell wall polysaccharides (like glucans) and the storage compound trehalose or transferred into other monosaccharides or their derivatives like alditols. Not surprisingly, this pathway was also regulated by AtfA at AcuF phosphoenolpyruvate carboxykinase [221] and AcuG fructose-1,6-bisphosphatase [222]. Importantly, AN3433 encodes an AtfA-controlled *SIP4* orthologous gene [104], which may take part in the regulation of gluconeogenesis [223].

Metabolisms of some other carbohydrates including gluconic acid (GukA gluconokinase), *N*-acetyl-D-glucosamine (AN1428 *N*-acetylglucosamine-6-phosphate deacetylase [162,224]), galactose (GalD encoding a galactose-1-phosphate uridylyl transferase and functioning in the Leloir pathway [225,226]), and mannose (AN1715 mannose-6-phosphate isomerase [180,181]) were also regulated by AtfA via binding to the promoter regions of the genes indicated in parentheses.

Outstandingly, downstream of glycolysis, the TCA cycle was under clear AtfA control at CitA citrate synthase, which is needed for conidial germination [227] and possesses putative STRE-s in its promoter [228], the iron-containing AcoA aconitase hydratase [229], and at IdpA isocitrate dehydrogenase [188,227] (Figure 8). Furthermore, the glyoxylate cycle was also regulated by AtfA at AcuD isocitrate lyase [221]. It is noteworthy that in the citric acid producer *Aspergillus niger*, transcripts of the *acuD* and *acuE* (coding for malate synthase; Figure 8) were more abundant in dormant conidia than in conidia after 1 h incubation in liquid medium [230]. In unstressed *A. nidulans* cultures, deletion of *atfA* resulted in the upregulation of *aclB* coding for ATP-citrate synthase (ATP-citrate lyase), fueling biosyntheses of lipids, steroids, and other compounds with cytoplasmic acetyl-CoA and supporting conidiogenesis [227].

Glycerol temporarily accumulates during germination [45] and may also be present in developing and resting conidia in low quantities [44] (Table 1). Fine-tuning of glycerol metabolism is therefore of paramount importance in both conidiogenesis and germination of conidia. Not surprisingly, some major glycerin metabolic enzymes are under AtfA control including the catabolic GcnA glycerol kinase [231,232], AN10499 NADP^+^-specific glycerol dehydrogenase [185,190], and AN1396 FAD-dependent glycerol 3-phosphate dehydrogenase maintaining redox potential across the mitochondrial inner membrane [231,232]. The glycerol biosynthetic GldB NADP^+^-dependent glycerol dehydrogenase is required for the osmotolerance of the fungus, and germination of mutant conidia was delayed under NaCl stress [170].

Considering the utilization of other carbon sources, AtfA also regulates the uptake and metabolism of acetate (Figure 8), an easily metabolizable compound for Aspergilli [233,234,235]. The strict AtfA-dependent control of acetate metabolism (Figure 8) indicates that resting *A. nidulans* conidia are prepared to grow on this carbon source after germination. AtfA-controlled genes include *acpA* and *facA* coding for a high-affinity acetate permease [233,234] and acetyl-CoA synthase [236], respectively, as well as *facC* (cytoplasmic [237,238]) and *acuJ* (mitochondrial and peroxisomal [238])-encoding carnitine *O*-acetyltransferases. The *coaT* gene with an AtfA-bound promoter encodes an acetyl-CoA hydrolase/CoA transferase which transfers CoA from toxic propionyl-CoA to acetate [239]. The acetate-inducible *aciA* putative formate dehydrogenase gene [188,240,241] was also under AtfA control, and this oxidoreductase may supply germinating conidia with NADH-reducing power [242].

Lipid metabolism of *A. nidulans* is also regulated by AtfA at some important genes, including *plcA*, encoding a putative phosphoinositide-specific phospholipase C, which plays important roles in the maintenance of hyphal growth, conidiation, conidiospore germination (especially at lower temperatures), and nuclear division [243]. PlcA also contributes to the sensing of high molecular mass carbon sources like polypectate [244]. Lipid synthesis is under AtfA control at the AN0981 long-chain fatty acid elongase [134].

AtfA also clearly governed the expression of a number of oxidoreductases including the AN6835 putative chytochrome P450 enzyme (CYP505A8) gene [107,245], which was found to be fludioxonil-responsive [77]. This oxidoreductase may take part in the degradation of plant ω-hydroxy fatty acids [107]. Interestingly, three putative alcohol dehydrogenase genes, AN2470, AN5355, and AN8628 [185], were also under direct AtfA control, among which AN2470 was responsive to nitrosative stress [181]. It is noteworthy that the quantity of AN9002 oxidoreductase decreased after 30 min germination of conidia [183], and AN6274 putative short-chain dehydrogenase gene was profoundly upregulated during early vegetative growth of *A. nidulans* [111].

Although only a small group of nucleoside metabolic genes were regulated by AtfA, *uapC* coding for a broad specificity purine permease [246], which is located in the plasma membrane [247,248], was under clear AtfA control. Interestingly, NH_4_^+^ ions induce the endocytotic internalization of UapC permease [249]. It is noteworthy that ArtA arrestin-like protein is connected to the ubiquitination and endocytosis of UapA uric acid-xanthine transporter, and ArtB arrestin-like protein, whose expression is under AtfA control, may also interact with some nucleobase transporters [250]. ArtB may function in the endocytotic fine regulation of nitrate and secondary carbon source transporters [207]. The AN6856 gene coding for a putative equilibrative nucleoside transporter protein [134], catalyzing bidirectional passive diffusion processes [246,251,252], is also under direct AtfA control.

Aspergilli typically produce a large number of hydrolases, including polysaccharide-decomposing enzymes, proteases, phytases, and phosphatases, which is a characteristic feature of their saprophytic lifestyle [253]. The presence of hydrolases in resting conidia may prepare these propagules either for germination in an environment with plant-derived biopolymers like hemicellulose as suitable carbon sources or for self-degradation [253]. In this study, the promoters of two genes coding for hemicellulose-degrading enzymes, the AN5231 exo-arabinanase and AN7781 alpha-L-arabinofuranosidase genes, were also bound by AtfA. These observations are in line with the plant biomass degrading potential of *A. nidulans* [254,255]. Interestingly, AN3835, an *A. niger inuR* orthologous gene [256], which regulates inulinolytic and sucrolytic genes in *A. niger* [257], was upregulated in the *A. nidulans* Δ*atfA* strain in unstressed cultures. Furthermore, increased expression of the *xtrE* putative xylose transporter gene [178] was also recorded in MSB-exposed cultures of the *atfA* gene deletion strain.

Considering protein degradation, AtfA-dependent control of AN6784 aspartyl protease [258] and AN7366 metallopeptidase may contribute to the degradation of proteins and the subsequent utilization of amino acids. Among the amino acid transporters, the expression of the AN1631 methionine permease [259] and *can1* arginine permease [260] genes were under AtfA control, and the expression of AN8990 encoding a putative GABA transporter [83,246] was also AtfA-dependent (Table S3).

Phospholipases under AtfA control included PlaA (cytosolic phospholipase A_2_ [261]), PldA (phospholipase D; positively responding to high osmolarity conditions [261,262]), and the putative phospholipase AN7604. Furthermore, locus AN4154, encoding an enzyme with putative 1-alkyl-2-acetylglycerophosphocholine esterase activity, and AN7524 putative lipase [121] were also among the AtfA targets.

## 4. Conclusions

This study provides the first comprehensive genome-wide map of AtfA binding in *Aspergillus nidulans* and reveals a fundamentally new mode of fungal transcriptional regulation. We show that AtfA occupies its target promoters constitutively, independent of oxidative stress, thereby pre-configuring conidia for rapid metabolic activation and stress resistance before encountering environmental challenges. By integrating chromatin occupancy with RNA-seq, metabolite profiling, and Δ*atfA* phenotypes, we demonstrate that AtfA synchronizes asexual development with primary metabolism, MAPK signaling, antioxidant defense, storage carbohydrate biosynthesis, light-response pathways, and cell membrane organization. These findings define a unifying regulatory architecture in which AtfA acts as a master coordinator of fungal developmental and physiological preparedness. Given the conservation of Atf1 orthologs across fungi, this anticipatory transcriptional strategy may represent a widespread mechanism with implications for fungal ecology, stress biology, pathogenicity, and industrial performance.

Our findings highlight a compelling but still incomplete picture. The upstream signals that activate constitutively bound AtfA have not yet been defined, and this aspect of regulation is still speculative. We hope our study provides a foundation for future work aimed at identifying these environmental cues and signaling events and clarifying how promoter-bound AtfA becomes transcriptionally active during conidiation and germination.

## Figures and Tables

**Table 1:** Conidiospore production, trehalose, erythritol, glycerol, and mannitol content of conidiospores as well as glucose yield in the surface cultures of the atfA::3XFLAG strain.

	Culture Condition			
	Untr. (2 d)	MSB Tr. (2 d)	Untr. (3 d)	MSB Tr. (3 d)
**Spore yields (10^6^/cm^2^)**	1.9 ± 0.3	0.8 ± 0.4 *	2.0 ± 0.3	1.7 ± 0.3
**Remaining glucose concentration in culture medium (g/L)**	0	1.8 ± 0.7	0	0
**Trehalose (pg/spore)**	0.15 ± 0.05	0.17 ± 0.01	1.3 ± 0.2 ###	1.1 ± 0.1 ###
**Erythritol (pg/spore)**	0.03 ± 0.02	0.17 ± 0.02 **	0.003 ± 0.002 ###	0.011 ± 0.004 ###, ***
**Glycerol (pg/spore)**	0.04 ± 0.02	0.043 ± 0.002 *	0	0.008 ± 0.007 ##
**Mannitol (pg/spore)**	0.25 ± 0.04	1.4 ± 0.2 **	0.87 ± 0.05 ###	1.35 ± 0.06 **

Data are presented as mean ± SD values calculated from three independent experiments. Significant difference between untreated and MSB treated cultures (***, *p* < 0.1%; **, *p* < 1%; *, *p* < 5%,) as well as 2 d and 3 d cultures within given treatment (### *p* < 0.1%; ## *p* < 0.5%) are indicated.

**Table 2:** Overlap of ChIP-seq peaks under different conditions (replicate overlaps and inter-condition overlaps expressed in percent).

	Untr. (2 d) Rep1	Untr. (2 d) Rep2	Untr. (3 d) Rep1	Untr. (3 d) Rep2	MSB Tr. (3 d) Rep1	MSB Tr. (3 d) Rep2
**Untr. (2 d) rep1**	100.00	68.29	72.52	75.48	79.28	79.92
**Untr. (2 d) rep2**	73.04	100.00	76.04	76.96	80.18	73.27
**Untr. (3 d) rep1**	72.09	69.34	100.00	76.11	74.63	70.19
**Untr. (3 d) rep2**	79.38	74.06	80.49	100.00	84.48	79.38
**MSB Tr. (3 d) rep1**	79.69	74.83	75.72	81.90	100.00	84.99
**MSB Tr. (3 d) rep2**	84.05	71.07	74.26	80.18	88.61	100.00

**Table 4:** Light-responsive genes possessing AtfA-bound promoter showing altered expression in Δ*atfA* compared with wild-type.

Gene ID	Gene Name	Functional Description	References	AtfA and AtfB Dependent Regulations (Kocsis et al., 2023) [5]	
				Unstressed Culture ^‡^	MSB-Exposed Culture ^‡^
AN1052	*veA* ^+^	Involved in light-sensitive control of differentiation and secondary metabolism	Kim et al., 2002; Bayram et al., 2008a [105,106]		
AN0709	*silG*	Putative zinc finger protein	Martins et al., 2014 [107]	**A-B**	**AA**
AN0045		Transcript induced by light in developmentally competent mycelia	Ruger-Herreros et al., 2011; Suzuki et al., 2013; Reese et al., 2021 [44,99,108]	**AA**	**AA**
AN0693		Transcript induced by light in developmentally competent mycelia	Ruger-Herreros et al., 2011; Suzuki et al., 2013; Reese et al., 2021 [44,99,108]	**AA**	**AA**
AN9285	*ccgA*	Induced by light; ortholog of glucose-repressible protein Grg1, putative	Ruger-Herreros et al., 2011; Suzuki et al., 2013 [44,99]	**AA**	**A-B**
AN5015	*conJ*	Induced by light	Suzuki et al., 2013 [44]	**A-B**	**A-B**
AN8640	*conF*	Induced by light	Suzuki et al., 2013 [44]	**AA**	**AA**
AN3361	*nopA*	Encoding a fungal opsin, induced by light	Ruger-Herreros et al., 2011; Yu et al., 2021 [99,101]	**AA**	**AA**
AN0387	*cryA*	coding for a deoxyribodipyrimidine photo-lyase; sensing UVA and blue light	Bayram et al., 2008b [109]	**AA**	**AA**
AN0604		Homolog of *S. pombe uve1* DNA repair endonuclease	Fraser et al., 2003 [110]	**AA**	**AA**
AN8638	*cetJ*	Conidia-enriched transcript J; hemerythrin domain-containing protein	Suzuki et al., 2013; Ruger-Herreros et al., 2011; Breakspear and Momany, 2007 [44,99,111]	**AA**	**AA**
AN5004		Transcript induced by light in developmentally competent mycelia	Suzuki et al., 2013; Ruger-Herreros et al., 2011 [44,99]	**A-B**	**AA**
AN5764		Transcript induced by light in developmentally competent mycelia	Suzuki et al., 2013; Ruger-Herreros et al., 2011 [44,99]	**AA**	**A-B**
AN8018		Transcript induced by light in developmentally competent mycelia; auxin efflux transporter family protein	Suzuki et al., 2013; Ruger-Herreros et al., 2011; Martins et al., 2014 [44,99,107]	**AA**	**A-B**
AN8339		Transcript induced by light in developmentally competent mycelia	Suzuki et al., 2013; Ruger-Herreros et al., 2011 [44,99]	**AA**	**AA**
AN8641		Transcript induced by light in developmentally competent mycelia	Suzuki et al., 2013; Ruger-Herreros et al., 2011 [44,99]	**A-B**	**AA**

^‡^—AA: gene regulated putatively by AtfA but not by AtfB; A-B: gene regulated putatively by both AtfA and AtfB, deletion of *atfA* and/or *atfB* reduces only partially the gene activity, based on transcriptomics data (Kocsis et al., 2023) [5].

## Data Availability

ChIP-seq data were deposited into the Gene Expression Omnibus database under accession number GSE306765 and are available at the following URL: https://www.ncbi.nlm.nih.gov/geo/query/acc.cgi?acc=GSE306765 (accessed on 29 August 2025). Protein structure data are available in Table S5. Transcriptomics data are available with the GSE220052 accession number of the Gene Expression Omnibus database (GEO; http://www.ncbi.nlm.nih.gov/geo/, accessed on 25 January 2023). The data that support the findings of this study are available from the corresponding authors upon reasonable request.

## Supplementary Materials

The following supporting information can be downloaded at: https://www.mdpi.com/article/10.3390/cells14241965/s1, Table S1: *Aspergillus nidulans* Stress Protein Database. Table S2: ChIP-seq data analysis. Table S3: *A. nidulans* genes with AtfA-bound promoters. Table S4: GO enrichment analysis of *A. nidulans* genes with AtfA binding sites in their promoters. Table S5: Structural origin of the proteins displayed with 3D structures in Figure 5, Figure 6, Figure 7 and Figure 8. Figure S1: Localizations of ChIP-seq reads in the upstream region of protein coding genes. In the upper panel, the localization of all reads is shown relative to gene promoters. The lower panel displays all gene promoters, with the reads present indicated in red. Figure S2: AtfA is involved in the orchestration of cellular biological processes. Biogenesis, maintenance and function of subcellular organelles (mitochondria, ER, eisosomes, vacuoles, autophagosomes), cytoskeletal elements, as well as the transcription and translation machineries are presented (more details are available in Table S3, Sheet 6). Figure was created using Biorender (https://biorender.com/). Figure S3: Phenotype check of the THS30.3 (wild-type), Δ*atfA* mutant and *atfA*::3XFLAG mutant in the presence of *t*BOOH, MSB and H_2_O_2_. Figure S4: Enriched GO categories. Visualization of selected GO enrichment results listed and highlighted in Table S4. The analysis was based on 502 genes containing AtfA binding sites (AtfA-BS set), tested against a background of 10,670 loci with at least one GO annotation, out of 10,952 total loci. Enrichment was assessed using a Fisher’s exact test combined with Benjamini–Hochberg false discovery rate (FDR) correction. Categories significantly enriched (FDR < 0.05) were filtered, and those most relevant to the study were selected for visualization.
