# Supplementary material for: Genome-Wide Mapping Reveals an Extensive AtfA Regulatory Influence on Development, Metabolism, and Stress Preparedness in Aspergillus nidulans
Source: Cells. 2025 Dec 10;14(24):1965. doi: 10.3390/cells14241965 (PMC12731236; doi:10.3390/cells14241965)
Supplement: Supplementary file 1 [file cells-14-01965-s001.zip › cells-3923599-supplementary/Supplementary Figure S1_R3.pdf]

Supplementary Figure S1

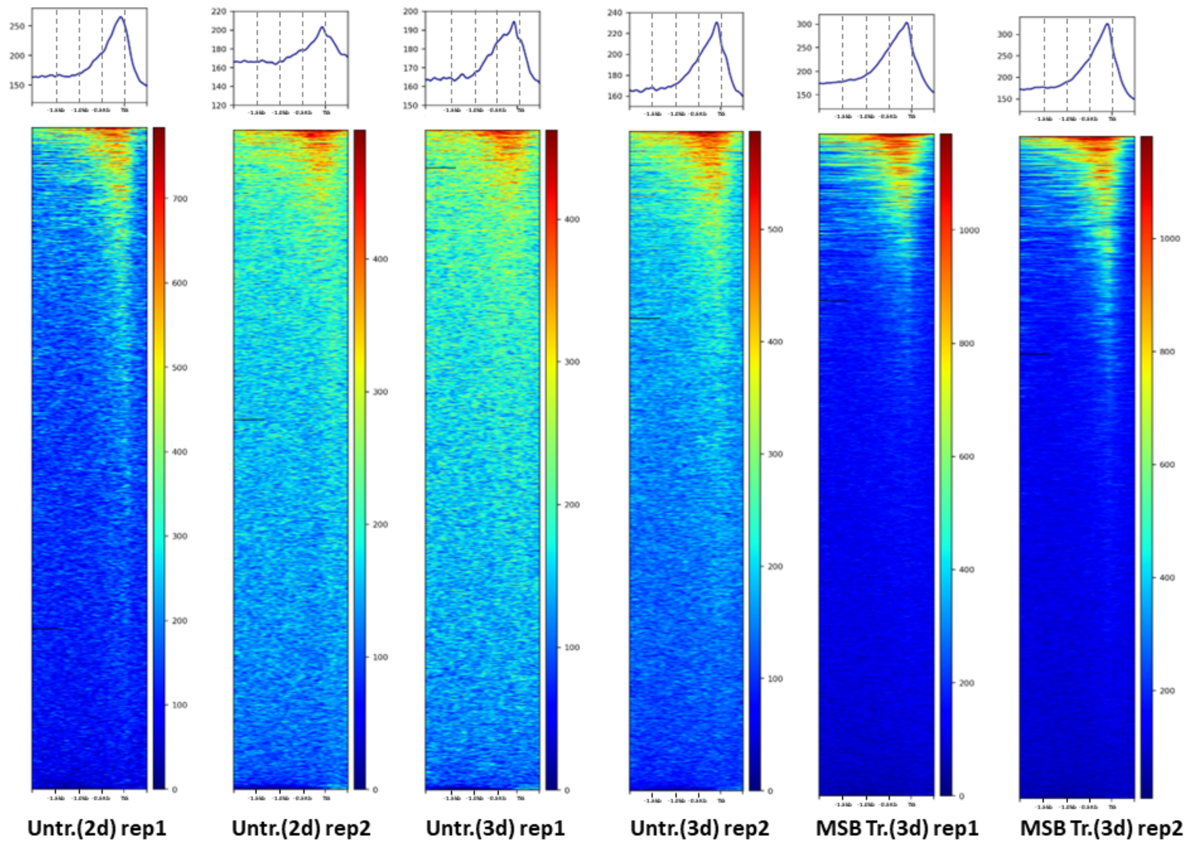

Figure S1. Localizations of ChIP-seq reads in the upstream region of protein coding genes. In the upper panel, the localization of all reads is shown relative to gene promoters. The lower panel displays all gene promoters, with the reads present indicated in red.
