# Supplementary material for: Genome-Wide Mapping Reveals an Extensive AtfA Regulatory Influence on Development, Metabolism, and Stress Preparedness in Aspergillus nidulans
Source: Cells. 2025 Dec 10;14(24):1965. doi: 10.3390/cells14241965 (PMC12731236; doi:10.3390/cells14241965)
Supplement: Supplementary file 1 [file cells-14-01965-s001.zip › cells-3923599-supplementary/Supplementary Figure S2_R3.pdf]

Supplementary Figure S2

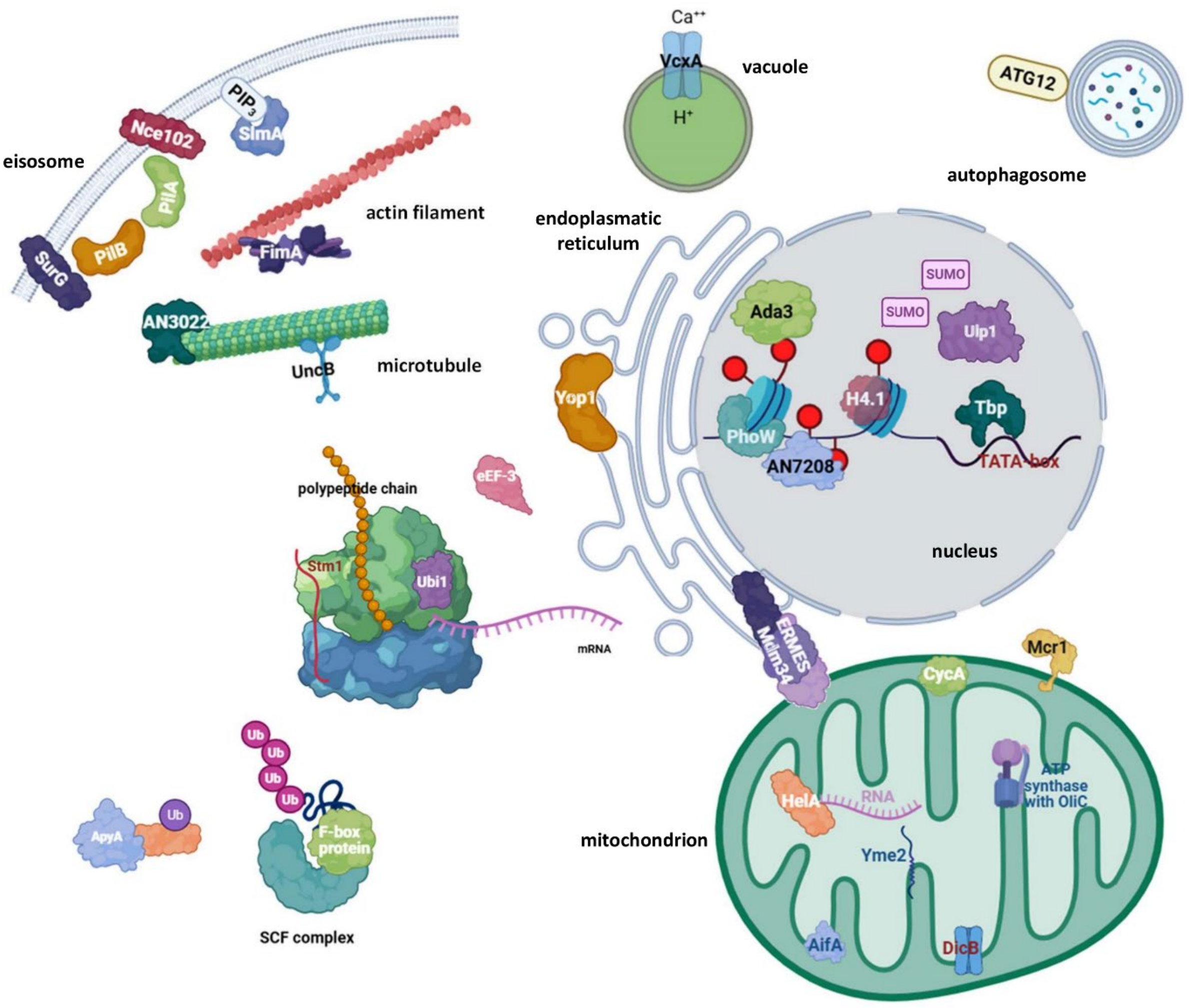

Figure S2. AtfA is involved in the orchestration of cellular biological processes. Biogenesis, maintenance and function of subcellular organelles (mitochondria, ER, eisosomes, vacuoles, autophagosomes), cytoskeletal elements, as well as the transcription and translation machineries are presented (more details are available in Table S3, Sheet 6). Figure was created using Biorender (https://biorender.com/).
