# Supplementary material for: Genome-Wide Mapping Reveals an Extensive AtfA Regulatory Influence on Development, Metabolism, and Stress Preparedness in Aspergillus nidulans
Source: Cells. 2025 Dec 10;14(24):1965. doi: 10.3390/cells14241965 (PMC12731236; doi:10.3390/cells14241965)
Supplement: Supplementary file 1 [file cells-14-01965-s001.zip › cells-3923599-supplementary/Supplementary Figure S3_R3.pdf]

Supplementary Figure S3

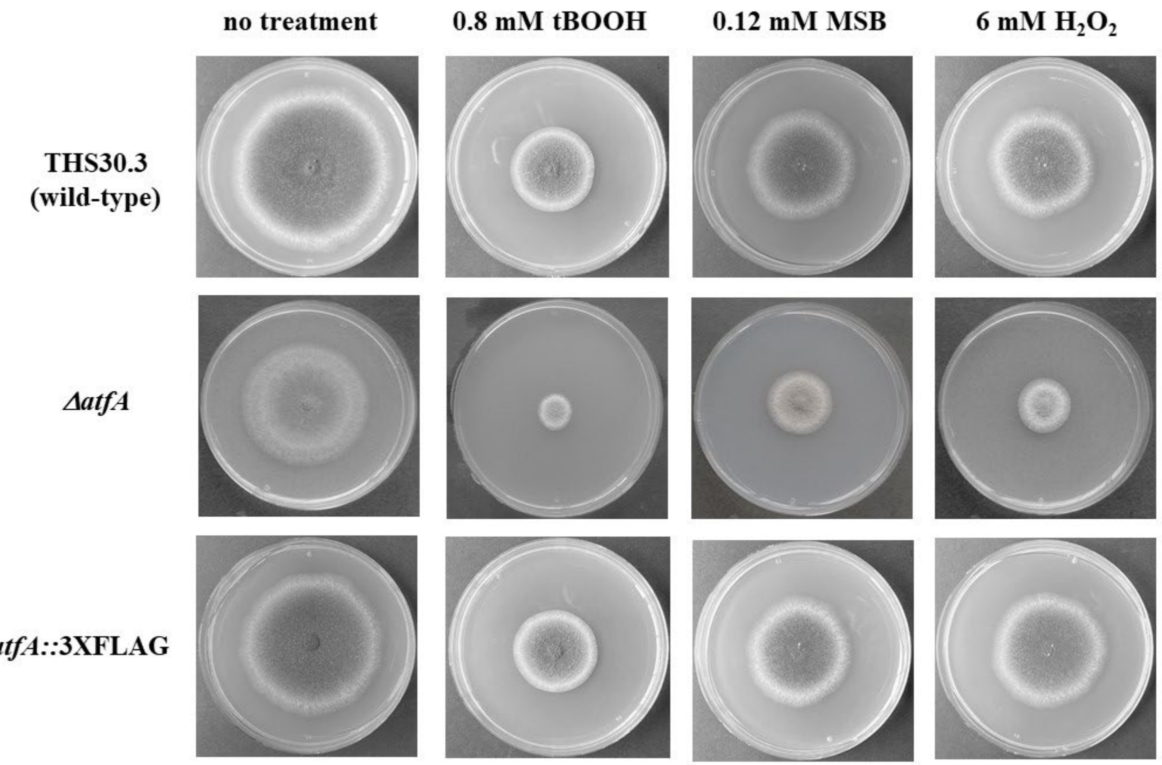

Figure S3. Phenotype check of the THS30.3 (wild-type), *ΔatfA* mutant and *atfA*::3XFLAG mutant in the presence of *t*BOOH, MSB and H_2O_2.
