# Supplementary material for: Genome-Wide Mapping Reveals an Extensive AtfA Regulatory Influence on Development, Metabolism, and Stress Preparedness in Aspergillus nidulans
Source: Cells. 2025 Dec 10;14(24):1965. doi: 10.3390/cells14241965 (PMC12731236; doi:10.3390/cells14241965)
Supplement: Supplementary file 1 [file cells-14-01965-s001.zip › cells-3923599-supplementary/Supplementary Figure S4_R3.pdf]

Supplementary Figure S4

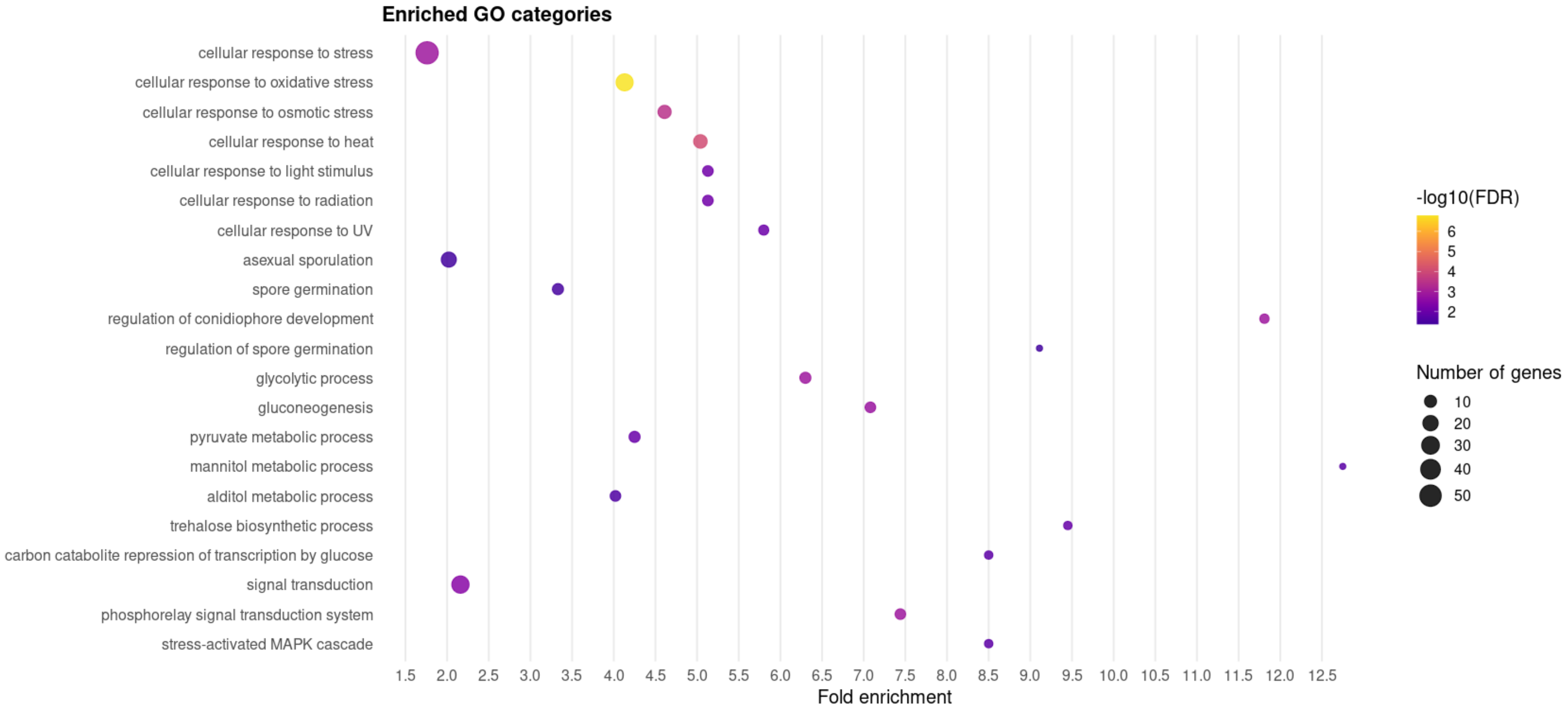

Figure S4. Enriched GO categories. Visualization of selected GO enrichment results listed and highlighted in Table S4. The analysis was based on 502 genes containing AtfA binding sites (AtfA-BS set), tested against a background of 10,670 loci with at least one GO annotation, out of 10,952 total loci. Enrichment was assessed using a Fisher's exact test combined with Benjamini–Hochberg false discovery rate (FDR) correction. Categories significantly enriched (FDR < 0.05) were filtered, and those most relevant to the study were selected for visualization.
